# Deterministic column subset selection for single-cell RNA-Seq

**DOI:** 10.1371/journal.pone.0210571

**Published:** 2019-01-25

**Authors:** Shannon R. McCurdy, Vasilis Ntranos, Lior Pachter

**Affiliations:** 1 California Institute for Quantitative Biosciences, University of California Berkeley, Berkeley, California, United States of America; 2 Department of Electrical Engineering and Computer Sciences, University of California Berkeley, Berkeley, California, United States of America; 3 Division of Biology and Biological Engineering, Department of Computing and Mathematical Sciences, California Institute of Technology, Pasadena, California, United States of America; Texas A&M University, UNITED STATES

## Abstract

Analysis of single-cell RNA sequencing (scRNA-Seq) data often involves filtering out uninteresting or poorly measured genes and dimensionality reduction to reduce noise and simplify data visualization. However, techniques such as principal components analysis (PCA) fail to preserve non-negativity and sparsity structures present in the original matrices, and the coordinates of projected cells are not easily interpretable. Commonly used thresholding methods to filter genes avoid those pitfalls, but ignore collinearity and covariance in the original matrix. We show that a deterministic column subset selection (DCSS) method possesses many of the favorable properties of common thresholding methods and PCA, while avoiding pitfalls from both. We derive new spectral bounds for DCSS. We apply DCSS to two measures of gene expression from two scRNA-Seq experiments with different clustering workflows, and compare to three thresholding methods. In each case study, the clusters based on the small subset of the complete gene expression profile selected by DCSS are similar to clusters produced from the full set. The resulting clusters are informative for cell type.

## Introduction

Advances in RNA sequencing technology have made it possible to measure the genome-wide expression profile of single cells [1]. This technology is not without computational and analytical challenges, some of which include quality control, quantification, normalization, technical variability, and other confounding factors such as batch effects [2, 3]. More general challenges stem from the high dimensionality of the expression profiles: for example, the challenge of selecting informative features from within the expression profiles.

One use for single-cell RNA sequencing (scRNA-Seq) data is the characterization of heterogeneity of expression within a population of cells for the discovery of new cell types through clustering of expression profiles [4]. This note explores the following question: is it possible reduce the number of features in the expression profile without a large effect on clustering and classification? This question is inspired by the quality control and technical variability challenges of scRNA-Seq. Common techniques for quality control and technical variability reduction include simple thresholding schemes and principal components analysis (PCA).

One commonly used technique to reduce the number of features in the data matrix involves selecting columns from the original data matrix **A**, to form a column submatrix **C**, by thresholding the individual columns based on a score. Workflows for scRNA-Seq such as *Seurat* [5], *Monocle* [6], *MAST* [7], *Cell Ranger* [8], *scater* [9], *scran* [10], and *SCANPY* [11] all include at least one such filtering steps. Frequently used scores are based on abundance or expression level [5, 6, 8–11], detection rates related to frequencies of zero values [6, 9–11], [7] (filtering described in supplement of [12]), and variance [6, 9]. We call these methods *simple column* thresholding methods, because the score for each column *i* depends only on column *i*. Furthermore, within each column *i*, covariance between the rows (cells) of that column is not taken into account. By selecting columns using simple column thresholding, and not linear combinations of columns from **A** as with PCA, the elements of **C** will maintain the properties of non-negativity, sparsity structure (e.g. the patterns of zeros in the retained columns), and interpretability. This is an advantage over PCA, but there are no guarantees that **C** will have other properties similar to the original data matrix **A** (e.g. a similar spectrum).

The rational behind these thresholding steps is that is that the most variable genes are responsible for the important differences between cells, and low-abundance genes and or genes with high dropout rates should be filtered out [10]. Currently there does not appear to be consensus in the literature on the best way to define a score for highly variable genes (see discussion in [10]). Scores to identify highly variable genes are based upon the coefficient of variation [13] or dispersion [5, 8, 11]. Some analysis workflows arrive at highly variable gene scores using heuristics such as binning in concert with thresholding [5, 8, 11], or modeling relationships between quantities such as the mean and variance [10]. It has also been argued that genes should be selected based on modeling the relationship between mean and dropout-rate [14]. These heuristics do not qualify as simple column thresholding and also do not come with guarantees.

Replacing the original data matrix of scRNA-Seq expression profiles with a rank-*k* PCA truncation of the profiles is another commonly used technique to reduce the number of features and the technical variability [3]. To understand the PCA truncation, we must establish some matrix notation that we will use throughout this note. We orient the original data matrix **A** so that the *n* rows are cells and *d* columns are features, where *n* < *d*. For PCA, singular value decomposition (SVD) is performed on the column-mean centered matrix $\tilde{A}=A-1\mu^{T}$, where **1** is an *n* × 1 column vector and $\mu =\frac{1}{n}A^{T}1$ is a *d* × 1 column vector of column-means. The sum of the spectrum of eigenvalues of $\tilde{A}{\tilde{A}}^{T}$ is proportional to the total empirical variance of **A**. The rank-*k* PCA truncation of **A**, which we call ${\tilde{T}}_{k}$, is the rank-*k* SVD truncation of $\tilde{A}$. SVD is reviewed in Section B in S1 File, and the formula for ${\tilde{T}}_{k}$ is provided there. As a consequence of the SVD, the spectrum of the square of the rank-*k* PCA truncation ${\tilde{T}}_{k}$ is identical to the spectrum of the square of the mean-centered data matrix $\tilde{A}$ up to rank *k*; PCA gives a rank-*k* approximation to the mean-centered data $\tilde{A}$ that preserves the maximum empirical variance of **A**. PCA is performed to reduce technical variability under the assumption that the technical variation is primarily captured by the non-leading eigenvalues and eigenvectors of $\tilde{A}{\tilde{A}}^{T}$. The technical variability due to dropout requires additional sophistication to address, such as *SCDE/PAGODA* [15], *ZIFA* [16], or *CIDR* [17]. The drawback of replacing the original data matrix with the rank-*k* PCA truncation of the data that it fails to preserve non-negativity and sparsity structures present in the original data matrix, and the coordinates of projected cells are not interpretable in terms of single features. PCA alone does not provide a column subset.

The goal of column subset selection (CSS) is to extract from a matrix **A** a column submatrix **C** that conserves favorable properties, such as conditions on the spectrum of the column submatrix **C** [18]. Like the simple column thresholding methods, CSS maintains the properties of non-negativity, sparsity structure, and interpretability, and like PCA, CSS conserves favorable matrix properties. Similar to the simple column thresholding methods discussed above, each column has a score, however in CSS algorithms, the score for each column *i* also depends on all of the other columns. This dependence is what allows **C** to retain favorable properties not guaranteed by simple column thresholding or other heuristics. We will consider rank-*k* subspace leverage scores in this note. Leverage scores have been considered for regression diagnostics and outlier detection in statistics [19, 20] and were brought to prominence more recently in the context of randomized matrix algorithms [21]. The rank-*k* subspace leverage score *τ*_*i*_(**A**_*k*_) for the *i*^*th*^ column of **A** is,

$$\begin{array}{c} \tau_{i}\left(A_{k}\right)=a_{i}^{T}{\left(A_{k}A_{k}^{T}\right)}^{+}a_{i}, \end{array}$$ (1)

where the *i*^*th*^ column of **A** is an (*n* × 1)-vector denoted by **a**_*i*_, **M**^+^ denotes Moore-Penrose pseudoinverse of **M**, and **A**_*k*_ is the rank-*k* SVD approximation to **A**, defined in Section B in S1 File. The leverage score *τ*_*i*_(**A**_*k*_) can also be written as the solution to the following optimization problem,

$$\begin{array}{c} \tau_{i}\left(A_{k}\right)=\min||\hat{x}{\left|\right|}_{2}^{2}\;\text{s}.\text{t}.\;\hat{x}=\text{argmin}||A_{k}x-a_{i}{\left|\right|}_{2}^{2},\;x\in R^{d}. \end{array}$$ (2)

where ${\left||x|\right|}_{2}^{2}$ refers to the Euclidean (*L*_2_) norm of the vector **x** (see Section C in S1 File for the proof). The vector **x** measures how easily the column **a**_*i*_ can be written as a linear combination of the columns of **A**_*k*_. Eq 2 shows that leverage scores capture the importance of each column **a**_*i*_ in the column space of **A**_*k*_ and are sensitive to collinearity between columns. We illustrate this point with a toy example in Section The DCSS algorithm [22].

CSS algorithms select columns either with a random sampling procedure (such as in [21]) or a deterministic procedure. We showcase the deterministic CSS (DCSS) algorithm introduced by [22]. [22] show that for datasets with power-law decay in *τ*_*i*_(**A**_*k*_), DCSS will select a least-squares approximation for **A**, **CC**^+^ **A**, requiring fewer columns with the same accuracy than random sampling methods. One of the contributions of this note is a new bound for the spectrum of the square of **C** selected by DCSS projected onto the rank-*k* subspace that best approximates **A** (Eq 9). This bound means that, once both **C** and **A** are projected onto the rank-*k* subspace that best approximates **A**, **CC**^*T*^ is “close” to **AA**^*T*^. Or, in other words, **CC**^*T*^ can be thought of as an approximation to $A_{k}A_{k}^{T}$, in a way made precise in Section New bounds for DCSS. Another consequence is that the Frobenius norm of **C** is bounded (Eq 10). The Frobenius norm is a measure of the “size” of a matrix, so this bound provides confidence that the DCSS column matrix **C** is also similar in “size” to **A** and **A**_*k*_. In the event that DCSS is performed on a mean-centered matrix $\tilde{A}$, the Frobenius norm provides a measure of empirical variance. We also show a similar bound holds for random sampling (Eq 11), and under the assumption of power-law decay, DCSS requires fewer columns for the same error than random sampling.

In addition to the spectral bound, we present two case studies on two different scRNA-Seq experimental and analysis workflows to illustrate empirically the effect of thresholding features with DCSS compared to read count, variance, and index of dispersion (empirical variance/mean) on clustering and classification. The comparison to these three interpretable simple column thresholding schemes provides insight into the features selected by DCSS. To the best of our knowledge, this is the first time DCSS has been applied to scRNA-Seq data. Our interest is in principled column subset selection, so we do not empirically compare DCSS to SVD, PCA, or other commonly used dimensionality reduction techniques such as t-distributed stochastic neighbor embedding (t-SNE) [23] because, while these methods do provide dimensionality reduction, they do not select column submatrices.

The first case study is the genome-wide expression profiles of 3, 005 cells from the mouse cortex and hippocampus [4] and the clustering workflow of [24]. The second case is the genome-wide expression profiles of 4, 423 cells from mouse bone marrow [25] and the trajectory workflow of [26]. We showcase our method on two different experimental and analysis workflows to illustrate the general utility of our method. The expression profile features in the first case study are derived from a partition of reads intermediate to gene expression quantification; the expression profile features in the second case study are gene unique molecular identifier (UMI) counts (see Section Results for DCSS for further discussion). These two different expression profile feature types have different collinearity and covariance properties, and so we illustrate the DCSS method with both types. For this note, we use previously studied analysis and clustering workflows that were tailored for the datasets at hand, since our purpose is to study the effects of DCSS and thresholding on clustering, and not to evaluate many different clustering algorithms or analysis workflows. Clustering is both an art and a science, and there are many different algorithms and workflows for clustering single-cell data; others not considered here include *CIDR* [17] and *SIMLR* [27].

In both case studies, DCSS reduces the low abundance genes and maintains many of the most variable and over-dispersed genes. This shows that DCSS shares the best features of the simple column thresholding methods and, like PCA, comes with additional bounds on the spectrum. This supports our conclusion that DCSS can be used instead of the simple column thresholding methods for quality control and to reduce technical variability, in addition to selecting informative features. In both case studies, only a small fraction of the features are necessary to obtain clusters reflecting cell types, consistent with results in [28]. We show that there is high similarity between the clustering assignments computed with the complete expression profile and the reduced expression profile.

## Methods

The aim of this note is to explore the effect of thresholding features (in our setting, measurements of gene expression) with DCSS. We compare DCSS to simple column thresholding methods and also to the complete data. These thresholding methods are often the first step in the pre-processing workflow. In this section, we include the DCSS algorithm for completeness, and we describe the new bounds for DCSS.

### The DCSS algorithm [22]

**Algorithm 1**. *The DCSS algorithm selects for the submatrix* **C** *all columns i with a rank-k subspace leverage score τ_i_*(**A**_*k*_) *above a threshold θ, determined by the error tolerance ϵ and the rank, k. The algorithm is as follows*.

1. *Choose the rank, k, and the error tolerance, ϵ*.
2. *For every column i, calculate the rank-k subspace leverage scores τ_i_*(**A**_*k*_) (Eq 1).
3. *Sort the columns by τ_i_*(**A**_*k*_), *from largest to smallest. The sorted column indices are π_i_*.
4. *Define an empty set* Θ = {}. *Starting with the largest sorted column index π*_0_, *add the corresponding column index i to the set* Θ, *in decreasing order, until*,
  $$\begin{array}{c} \sum_{i\in \Theta }\tau_{i}\left(A_{k}\right)>k-\epsilon , \end{array}$$ (3)
  *and then stop. Note that* $k=\sum_{i=1}^{d}\tau_{i}\left(A_{k}\right)$. *It will be useful to define* $\tilde{\epsilon }=\sum_{i\notin \Theta }\tau_{i}\left(A_{k}\right)$. Eq 3 *can equivalently be written as* $\epsilon >\tilde{\epsilon }$.
5. *If the set size* |Θ| < *k, continue adding columns in decreasing order until* |Θ| = *k*.
6. *The leverage score τ*_*i*_(**A**_*k*_) *of the last column i included in* Θ *defines the leverage score threshold θ*.
7. *Introduce a rectangular selection matrix* **S** *of size d* × |Θ|. *If the column indexed by* (*i*, *π*_*i*_) *is in* Θ, *then* $S_{i,\pi_{i}}=1$. $S_{i,\pi_{i}}=0$ *otherwise. The DCSS submatrix is* **C** = **AS**.

*This algorithm requires* O(min(n,d)nd) *arithmetic operations, and there are modifications to the algorithm that improve the runtime* [22].

Theorem 3 of [22] states that when the rank-*k* subspace leverage scores exhibit a power-law decay in the sorted column index *π*_*i*_,

$$\begin{array}{c} \tau_{\pi_{i}}\left(A_{k}\right)=\pi_{i}^{-a}\tau_{\pi_{0}}\left(A_{k}\right)\;\;a>1, \end{array}$$ (4)

the number of sample columns selected by DCSS is,

$$\begin{array}{c} \left|\Theta \right|=\max({\left(\frac{2k}{\epsilon }\right)}^{\frac{1}{a}}-1,{\left(\frac{2k}{(a-1)\epsilon }\right)}^{\frac{1}{a-1}}-1,k). \end{array}$$ (5)

[22] demonstrate the power-law decay behavior of many real-world datasets; we show that this behavior is a reasonable assumption for the scRNA-Seq applications in Section Results.

For a statistical interpretation of DCSS, consider the data **a**_*i*_, *i* = 1, …, *d* to be identically and independently distributed (i.i.d.) according to the degenerate multivariate distribution $N(0,A_{k}A_{k}^{T})$. See [29] pg. 527-528 for a discussion of the degenerate multivariate distribution. Then the total likelihood of the data matrix **A** is,

$$\begin{array}{cc} L(A) & =\frac{1}{{\left(2\pi \right)}^{\frac{1}{2}kd}\prod_{j=1}^{k}{\left|\sigma_{j}\right|}^{d}}\exp(-\frac{1}{2}\sum_{i=1}^{d}a_{i}^{T}{\left(A_{k}A_{k}^{T}\right)}^{+}a_{i})\\ & =\frac{1}{{\left(2\pi \right)}^{\frac{1}{2}kd}\prod_{i=j}^{k}{\left|\sigma_{j}\right|}^{d}}\exp(-\frac{1}{2}\sum_{i=1}^{d}\tau_{i}\left(A_{k}\right)), \end{array}$$ (6)

where |*σ*_*j*_| are the *k* largest singular values of **A**_*k*_. In contrast, the total likelihood of the DCSS matrix **C** is,

$$\begin{array}{cc} L(C) & =\frac{1}{{\left(2\pi \right)}^{\frac{1}{2}k\left|\Theta \right|}\prod_{j=1}^{k}{\left|\sigma_{j}\right|}^{\left|\Theta \right|}}\exp(-\frac{1}{2}\sum_{i\in \Theta }\tau_{i}\left(A_{k}\right))\\ & =\frac{1}{{\left(2\pi \right)}^{\frac{1}{2}k\left|\Theta \right|}\prod_{j=1}^{k}{\left|\sigma_{j}\right|}^{\left|\Theta \right|}}\exp(-\frac{1}{2}\sum_{i\in \Theta }\tau_{i}\left(A_{k}\right)-\frac{1}{2}\sum_{i\notin \Theta }\tau_{i}\left(A_{k}\right)+\frac{1}{2}\tilde{\epsilon })\\ & =L\left(A\right)\exp(\frac{1}{2}\tilde{\epsilon }){\left(2\pi \right)}^{\frac{1}{2}k\left(d-|\Theta |\right)}\prod_{j=1}^{k}{\left|\sigma_{j}\right|}^{d-|\Theta |}. \end{array}$$ (7)

This shows that the DCSS matrix **C** preserves the total likelihood of the data up to a factor of $\exp(\frac{1}{2}\tilde{\epsilon })<\exp(\frac{1}{2}\epsilon )$ and a normalization constant, under the assumption that the data is i.i.d. according to $N(0,A_{k}A_{k}^{T})$. Any other selection set Θ′ of the same number of columns (|Θ′| = |Θ|) will have equal or greater error (*ϵ* ≤ *ϵ*′). This interpretation illustrates that DCSS accounts for covariance $A_{k}A_{k}^{T}$ between rows (cells).

The DCSS method has two parameters, *k*, *ϵ* which jointly determine the number of columns |Θ| in the DCSS column submatrix **C**. The parameter *k* determines the rank of interest of the SVD approximation to **A**. The tuning parameter *ϵ* is a measure of the error tolerance in the “size” of **C** compared to **A**_*k*_. Given *k*, the desired error tolerance *ϵ* determines the number of columns |Θ|; completely equivalently, one could instead select the desired number of columns |Θ| and determine the resulting error tolerance *ϵ*. The parameters *k*, *ϵ*, and |Θ| will be different for different datasets and workflows. The selection of these parameters is a model selection problem, and in concert with a loss function, one could select these parameters using one’s preferred model selection method (e.g. cross-validation). The aim of this note, to compare clustering performed with the complete data matrix and a column submatrix, does not have a well-defined loss function, and so we use the heuristic “elbow” method for selecting *k* [30], and we choose *ϵ* to be 0.1 or 0.05, depending on the biological application.

As a toy example to illustrate how DCSS differs from the simple column thresholding methods, consider the following toy data matrix,

$$\begin{array}{c} A=(\begin{array}{ccc} 40 & 20 & 10\\ 20 & 10 & 15 \end{array}). \end{array}$$ (8)

If the goal is to select a column submatrix with two columns, it is easy to check that simple column thresholding by mean, variance, and index of dispersion all select the first and second columns. However, the resulting column submatrix is only rank 1, because the first and second columns are linearly dependent. In contrast, DCSS with (*k* = 2, *ϵ* > 0.2) will select the first and third columns, and the resulting DCSS column submatrix will be rank 2. Unlike the first three methods, DCSS takes into account the collinearity between columns in the selection procedure. If the DCSS error tolerance for this toy example is less than 0.2, DCSS will select all three columns.

We also mention two asides: first, in applications where the number of cells is far greater than the number of gene features (*n* > *d*), the method can instead be applied to **A**^*T*^ instead of **A** to filter cells instead gene features; second, the method can be modified to select columns for any rank-*k* subspace defined by *k* singular vectors of **A**, and not just the leading-*k* subspace (e.g. drop component 1 but include component 2). This could be useful when some of the leading singular vectors are highly correlated with batch, dropout, or other confounding effects.

### New bounds for DCSS

We derive a new spectral approximation bound (Eq 9) for the square of the submatrix **C** selected with DCSS and projected onto the rank-*k* subspace that best approximates **A**.

**Theorem 1**. *Let* $A\in R^{n\times d}$ *be a matrix of at least rank k and τ*_*i*_(**A**_*k*_) *be defined as in* Eq 1. *Construct* **C** *following the DCSS algorithm described in Section The DCSS algorithm* [22]. *Then* **C** *satisfies*,

$$\begin{array}{ccc} \left(1-\epsilon \right)A_{k}A_{k}^{T} & ⪯ & U_{k}U_{k}^{T}{\text{CC}}^{T}U_{k}U_{k}^{T}⪯A_{k}A_{k}^{T}. \end{array}$$ (9)

**U**_*k*_ *is the matrix of left singular vectors from the rank-k SVD approximation to* **A**, *defined in Section B in* S1 File. *The symbol* ⪯ *denotes the Loewner partial ordering which is reviewed in Section B in* S1 File. *Conceptually, the Loewner ordering is the generalization of the ordering of real numbers* (*e.g*. 1 < 1.5) *to Hermitian matrices*.

This bound means that after projection onto the rank-*k* subspace that best approximates **A**, **CC**^*T*^ is “close” to **AA**^*T*^ on that subspace. Statements of Loewner ordering are quite powerful; important consequences include inequalities for the eigenvalues and Euclidean distances. Some of the consequences of the Loewner ordering are reviewed in Section B in S1 File. Eq 9 and the fact that **CC**^*T*^ ⪯ **AA**^*T*^ implies a bound on the Frobenius norm of **C**, a measure of the “size” of a matrix,

$$\begin{array}{ccc} \left(1-\epsilon \right)||A_{k}{\left|\right|}_{F}^{2} & \leq & {\left||C|\right|}_{F}^{2}\leq {\left||A|\right|}_{F}^{2}. \end{array}$$ (10)

In the event that **A** is mean-centered, Eq 10 means that the total empirical variance of **C** is bounded from below by (1 − *ϵ*) the variance in **A**_*k*_ and bounded from above by the total variance of **A**, similar to PCA (discussed in Section Introduction). The proof of Eqs 9 and 10 is included in Section D in S1 File. To develop further intuition for these bounds, consider a mean-centered **A**. Consider also the PCA truncation ${\tilde{T}}_{k}$ (defined in Section B in S1 File) instead of a column matrix **C** in Eqs 9 and 10. The PCA truncation ${\tilde{T}}_{k}$ also satisfies both bounds.

One simple consequence of Eq 9 is the following bound,

$$\begin{array}{ccc} \left(1-\epsilon \right)A_{k}A_{k}^{T} & ⪯ & U_{k}U_{k}^{T}{\text{CC}}^{T}U_{k}U_{k}^{T}⪯\left(1+\epsilon \right)A_{k}A_{k}^{T}. \end{array}$$ (11)

Eq 11 also holds for **C** selected by random sampling methods with *t* columns (see Section E in S1 File for the theorem and proof). Thus, DCSS selects fewer columns with the same accuracy *ϵ* in Eq 11 for power-law decay in the rank-*k* subspace leverage scores when,

$$\begin{array}{c} \left|\Theta \right|=\max({\left(\frac{2k}{\epsilon }\right)}^{\frac{1}{a}}-1,{\left(\frac{2k}{(a-1)\epsilon }\right)}^{\frac{1}{a-1}}-1,k)<\frac{2}{\epsilon^{2}}\left(k+m\gamma \right)(1+\frac{1}{3}\epsilon )\ln(\frac{16k}{\delta })\leq t. \end{array}$$ (12)

In this expression, *m* is the number of columns with zero rank-*k* subspace leverage score, *γ* is the minimum non-zero leverage score, and *δ* is the probability that Eq 11 fails to hold under random sampling.

## Results

We present two case studies where we compare DCSS to the simple column thresholding methods of variance, count, and index of dispersion. We analyze the overlap in the selected columns. We also illustrate the effect of DCSS compared to the complete data for single-cell clustering.

### Mouse cortex and hippocampus single-cell gene expression

As a concrete illustration of the DCSS method, we focus on the genome-wide expression profiles of 3005 cells from the mouse somatosensory cortex and hippocampal CA1 region [4] and the clustering workflow of [24]. The main contribution of [24] is to perform clustering directly on the partition of reads into equivalence classes (ECs) rather than on a full quantification of reads into gene expression. ECs are a partition of reads into distinct classes, such that every read in a class maps to exactly the same set of transcripts [31]. This method is computationally scalable, comparable across scRNA-Seq experiments, and can be more accurate than clustering performed on a full quantification of reads into gene expression profiles [24].

**Fig 1:**
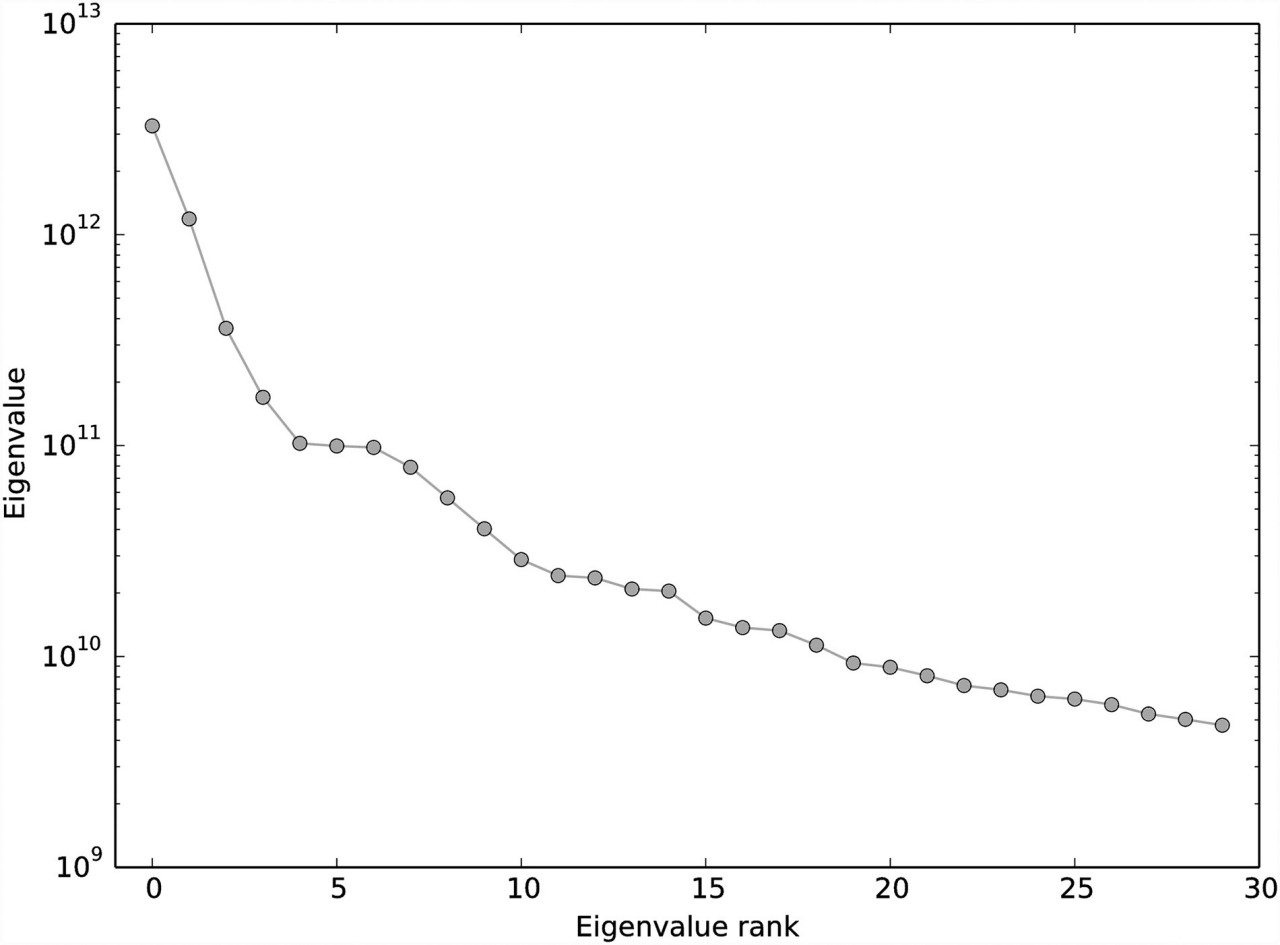
Eigenvalues for AA^*T*^, where A is the data matrix from the mouse cortex scRNA-Seq experiment [4] and the clustering workflow of [24]. The first “elbow” occurs at the fifth largest eigenvalue. We choose *k* = 5 for the DCSS workflow.

**Fig 2:**
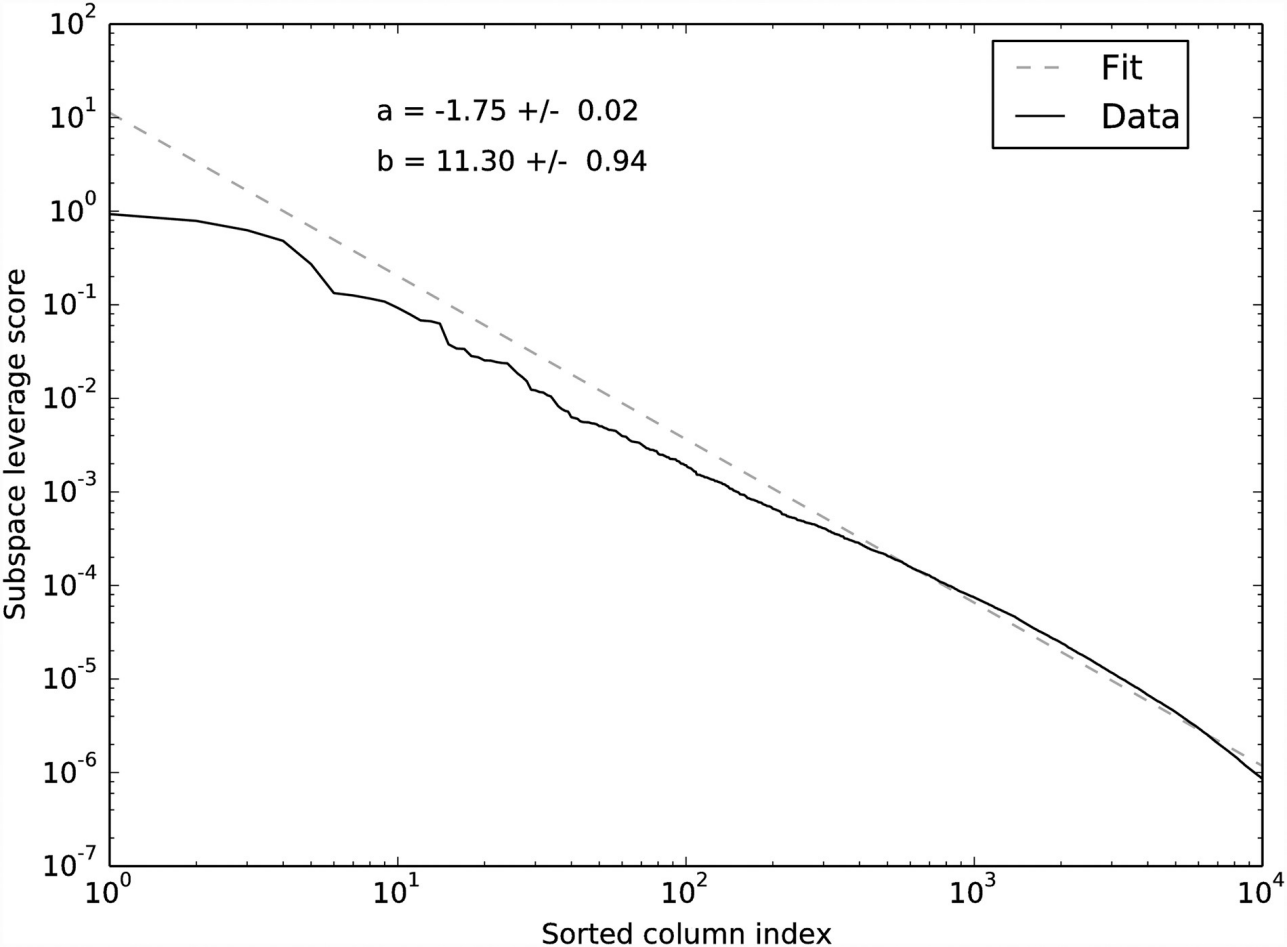
Power-law decay of *k* = 5 subspace leverage scores with sorted index for each column in A, where A is the data matrix from the mouse cortex scRNA-Seq experiment [4] and the clustering workflow of [24]. The fit is to Score = b × (Index)^*a*^. This shows that power-law decay is a reasonable assumption for this dataset.

The [24] data matrix **A** is 3, 005 cells × 246, 981 EC counts. By the elbow method, we choose *k* = 5 for the DCSS workflow (Fig 1). We select an error tolerance of *ϵ* = 0.1. The DCSS algorithm ran in less than a minute. The rank-5 subspace leverage scores and the power-law fit for the top-scored 10, 000 ECs are shown in Fig 2. The column submatrix **C** has only 862 ECs, or approximately 0.3% of the total ECs. These ECs contain 42.3% of the reads. These 862 ECs map to 2, 748 transcripts and to 1, 642 genes. Table 1 contains the gene ontology term enrichment analysis [32] on the genes corresponding to the DCSS (*k* = 5, *ϵ* = 0.1) ECs. Enrichments relevant for the brain include neuron part, neuron projection, and olfactory receptor activity. The enrichment analysis has an important caveat: because we map ECs to transcripts without positing an error model, there could be a high rate of false positives in the resulting transcripts and genes.

**Table 1:** PANTHER overrepresentation test (release 20160715) with the GO ontology database (release 2016-08-22) for the submatrix C selected from the data matrix A from the mouse cortex scRNA-Seq experiment [4] and the clustering workflow of [24] using DCSS with *k* = 5, *ϵ* = 0.1. The 862 DCSS-selected ECs of **C** are mapped to 1, 642 genes for the PANTHER overrepresentation test.

Type	Gene ontology (GO) term	Bonferroni p-value
Biological process	cellular component organization (GO:0016043)	1.12E-02
Biological process	cellular component organization or biogenesis (GO:0071840)	8.01E-03
Biological process	localization (GO:0051179)	4.37E-02
Cellular component	neuron projection (GO:0043005)	4.52E-04
Cellular component	neuron part (GO:0097458)	8.24E-05
Cellular component	cell projection (GO:0042995)	8.36E-03
Cellular component	cytoplasm (GO:0005737)	1.59E-02
Cellular component	intracellular part (GO:0044424)	4.89E-02
Molecular function	enzyme binding (GO:0019899)	3.35E-02
Molecular function	olfactory receptor activity (GO:0004984)	1.30E-02

**Fig 3:**
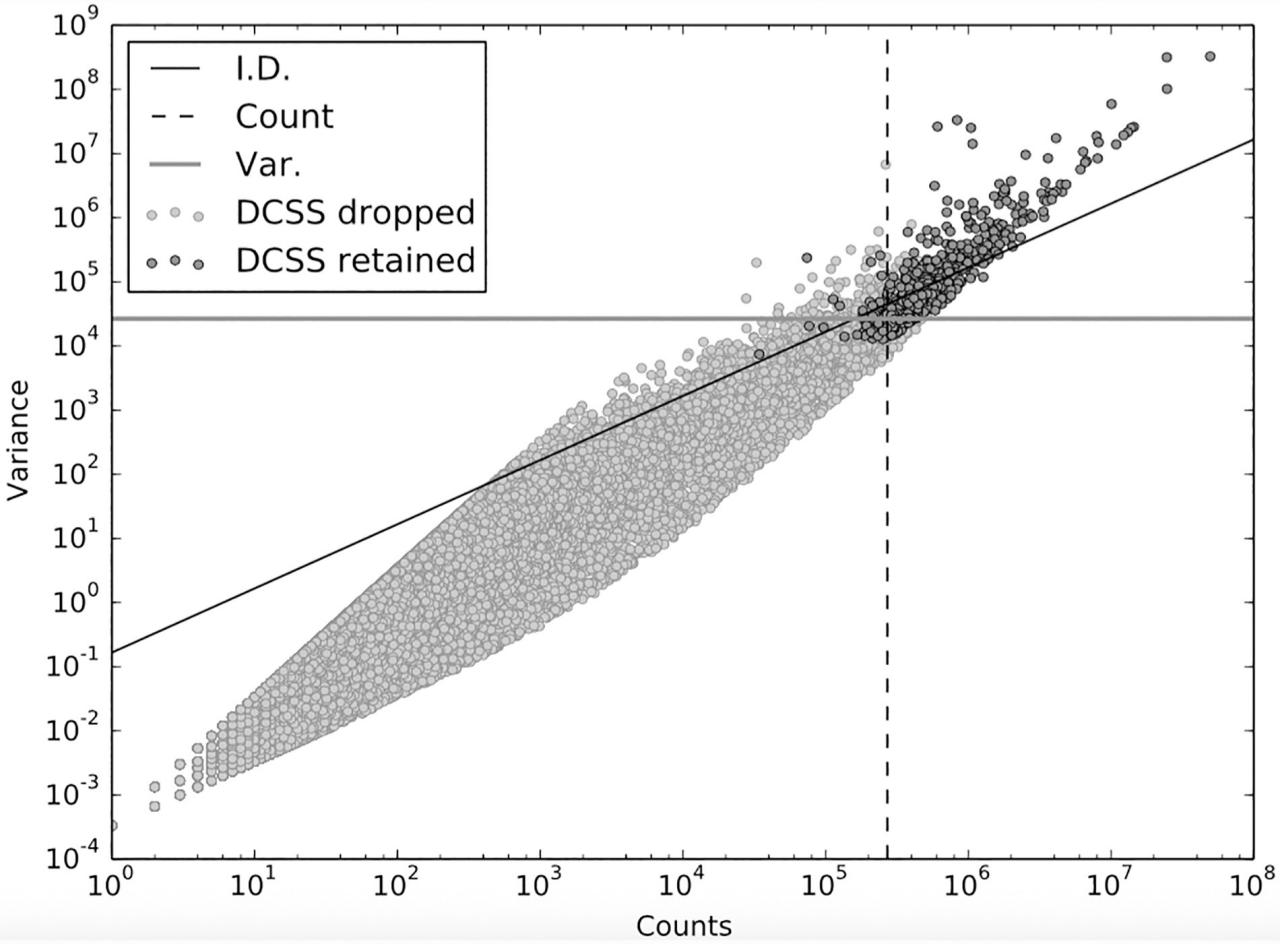
Count-variance plot for each column of A, where A is the data matrix from the mouse cortex scRNA-Seq experiment [4] and the clustering workflow of [24]. The color for each column represents whether the column is selected or not by *k* = 5, *ϵ* = 0.1 DCSS. The plot also shows the thresholds for count, variance, and index of dispersion with same number of selected columns as DCSS. The columns selected by DCSS are highly variable and have large counts.

**Fig 4:**
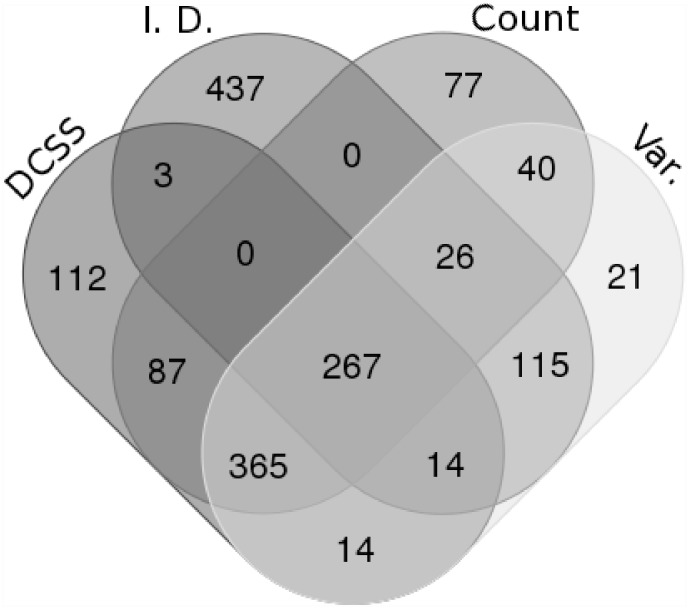
Venn diagram comparing the overlap between selected columns between *k* = 5, *ϵ* = 0.1 DCSS, count, variance, and index of dispersion thresholding on the data matrix from the mouse cortex scRNA-Seq experiment [4] and the clustering workflow of [24]. The four methods select many of the same columns. Recall from the toy example in Eq 8 that changing only one column can significantly change properties of the submatrix. Figure tool credit: [33].

We are interested in how differently selected subsets of columns of the same size compare, so we compare DCSS to the three simple column thresholding methods with the same number of columns in Figs 3 and 4. These figures show the similarities and differences in columns selected by the four thresholding methods. The simple column thresholding methods have sharp boundaries in Fig 3, while the DCSS boundary is not linearly separable. The DCSS boundary approximately interpolates between the count and variance boundaries, and is most distinct from the index of dispersion boundary. Fig 4 summarizes the overlap between selected columns in a Venn diagram. These figures illustrate that the DCSS method selects columns that are highly variable, have large counts, and frequently are over-dispersed; as such, the DCSS method is prescribed for quality control and to control technical variability.

The [24] workflow for the [4] dataset is to perform spectral clustering on pairwise Jensen-Shannon (JS) distances derived from the partition of reads into ECs. The spectral clustering algorithm used is standard; the algorithm is to perform *k*-means clustering on the *k*-dimensional SVD projection of the normalized Laplacian of the symmetric similarity matrix **S**. The similarity matrix used for spectral clustering is *S*(**p**, **q**) = 1 − *D*_*JS*_(**p**, **q**), where *D*_*JS*_(**p**, **q**) is the JS distance between two probability mass functions $p,q\in R^{d}$. JS distances are well-suited to high-dimensional data, and provide more accurate clustering than *L*_2_ distances on scRNA-Seq data [24]. For the [4] data, the probability mass function for each cell is the vector of EC counts, normalized to sum to one. For the four thresholded workflows (DCSS, count, variance, and index of dispersion), the probability mass function for each cell is the subset vector of EC counts, normalized to one.

**Fig 5:**
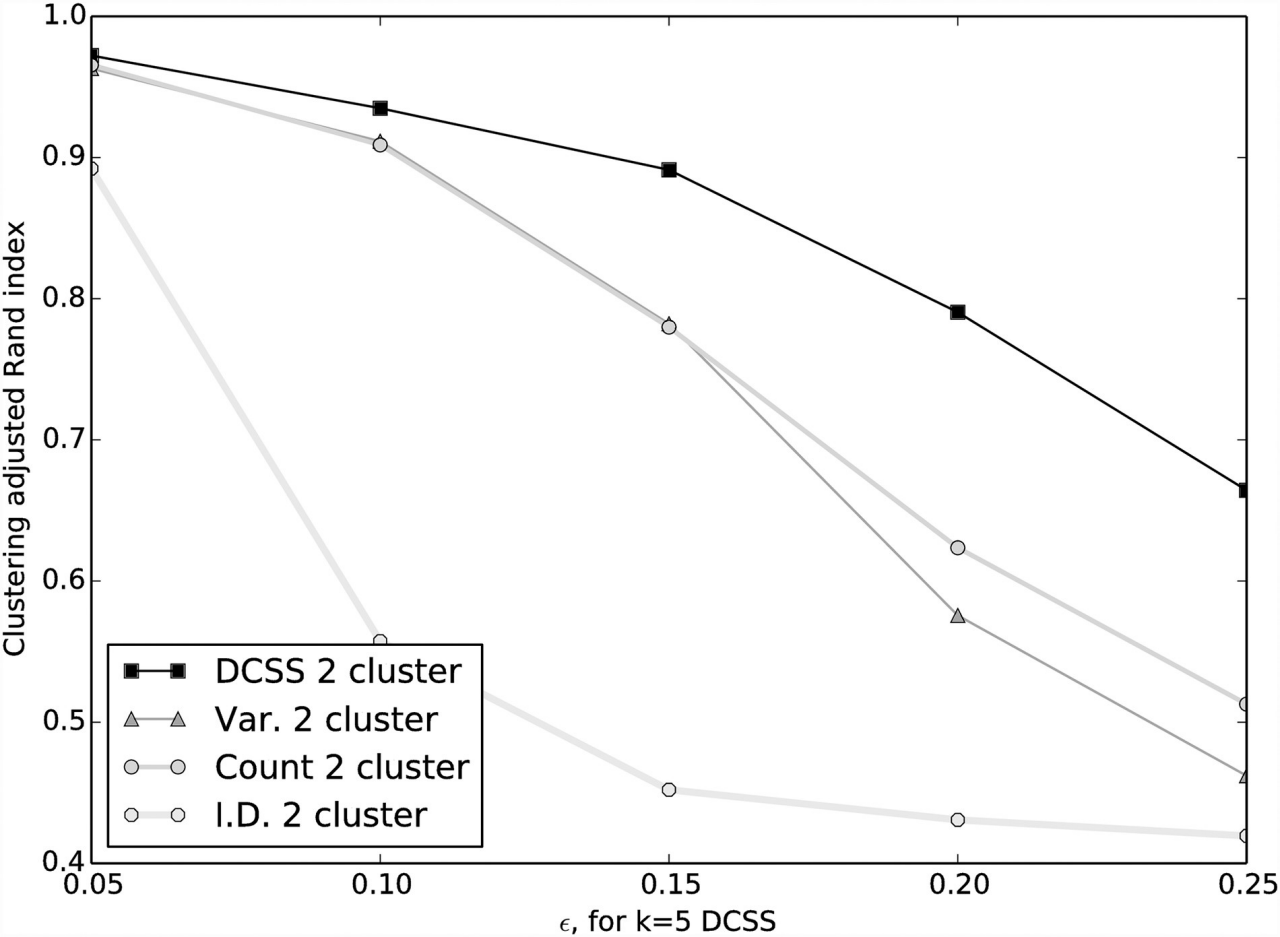
Average spectral clustering ARI for two clusters for DCSS, count, variance, and index of dispersion thresholding on the data matrix from the mouse cortex scRNA-Seq experiment [4] and the clustering workflow of [24]. Perfect agreement between cluster assignments has an ARI of 1. We vary the error tolerance *ϵ* with *k* = 5 for DCSS. Increasing the error tolerance decreases the agreement between clusters.

**Fig 6:**
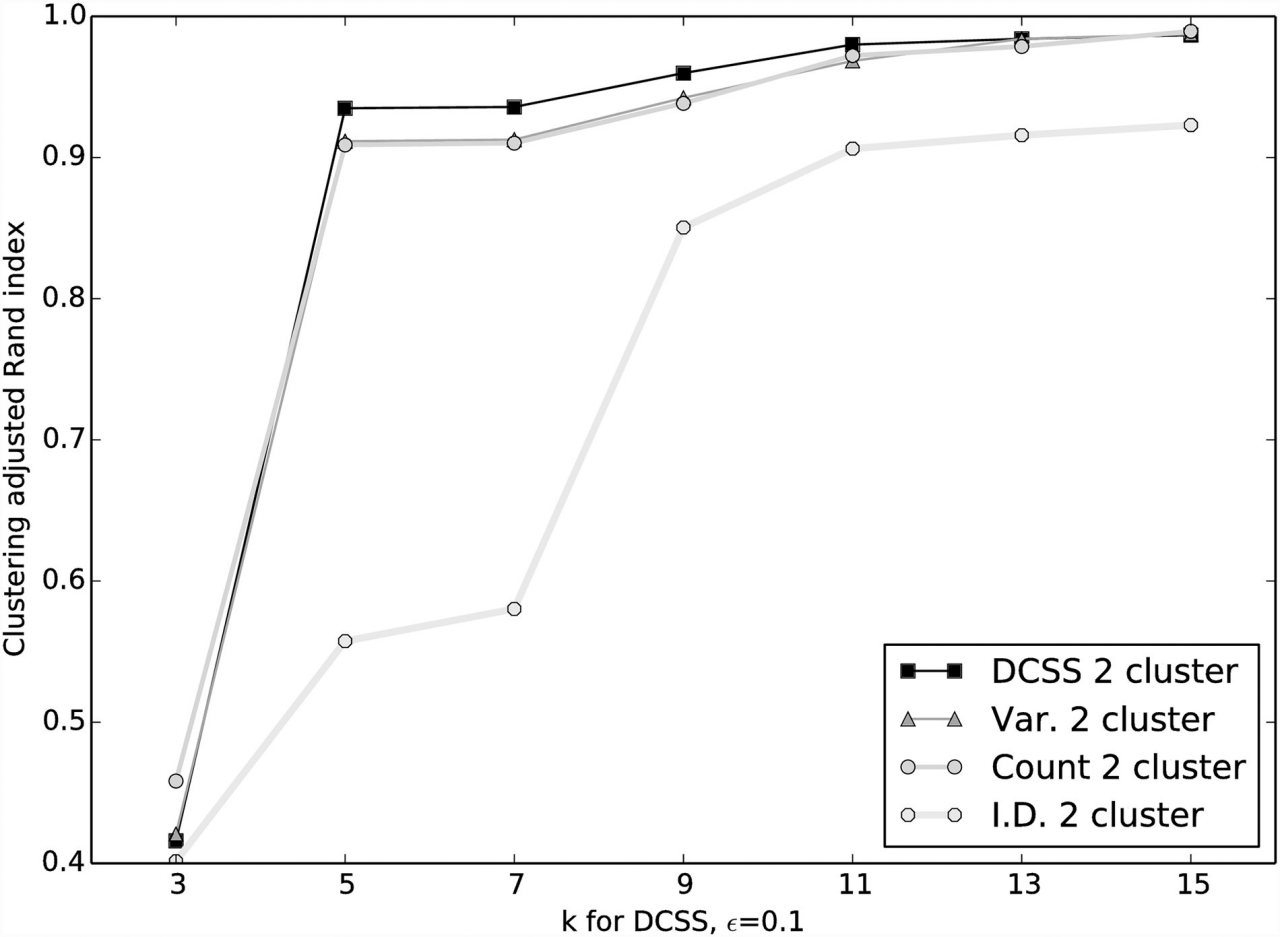
Average spectral clustering ARI for two clusters for DCSS, count, variance, and index of dispersion thresholding on the data matrix from the mouse cortex scRNA-Seq experiment [4] and the clustering workflow of [24]. We vary the dimension *k* with fixed error tolerance *ϵ* = 0.1 for DCSS. Increasing the dimension increases the agreement between clusters.

We evaluate the spectral clustering classification similarity using the adjusted Rand index (ARI) [34]. The ARI is a symmetric measure of similarity between two clustering assignments that counts the number of pairwise agreements between the two assignments, adjusted for chance. It takes values between −1 and 1. Perfect agreement between assignments has an ARI of 1, and random assignments have an expected ARI of 0. We compare the average ARI between the complete data and thresholded workflows, regarding the complete data workflow as the ground-truth. This is an imperfect measure, since the complete data has both noise and signal, but it is a reasonable measure for real biological data, since one does not have access to the ground-truth. Since spectral clustering requires a random initialization for *k*-means, the average is over *T* = 10 random initializations. Figs 5 and 6 show the average spectral clustering ARI for two spectral clusters for the workflow with the matrix **A** and the workflow with the column submatrix **C** for various *k*, *ϵ*. The S1 File contains similar figures for nine clusters (Figs A and B in S1 File). The different cells were curated into 47 subtypes by [4], but we evaluate our method on coarser-grained classifications because we have higher confidence in the spectral clustering ground-truth. Two spectral clusters identify neurons and non-neurons, while nine spectral clusters only loosely correspond to the nine major cell types. We also include the average ARI for the three simple column thresholding methods with the same number of columns as the DCSS method. We find that 0.3% of the total ECs give an average ARI of 0.93 compared to the complete data for two clusters for *k* = 5, *ϵ* = 0.1 DCSS; only a small fraction of the gene expression profiles currently produced in scRNA-Seq experiments may be necessary to obtain the clusters reflecting cell types.

### Mouse bone marrow single-cell gene expression

As a second application of the DCSS method, we focus on the genome-wide mRNA expression profiles of 4, 423 cells from mouse bone marrow myeloid progenitors [25], and the *wishbone* trajectory workflow of [26]. The contribution of [26] to scRNA-Seq is to use diffusion maps to identify components related to the development and maturation of cells, specifically myeloid and erythroid progenitors from hematopoietic stem and progenitor cells (HSPCs).

The [26] data matrix for the [25] dataset is **A** is 4, 423 cells × 14, 955 gene unique molecular identifier (UMI) counts. The [26] workflow is quite involved. In brief, the *wishbone* algorithm creates a nearest-neighbor Euclidean distance graph. This graph is used to estimate all of the shortest path distances between a set of randomly sampled cells and the rest of the cells, and the shortest path distances are used to make the trajectory and branch assignments. The *wishbone* algorithm acts on a set of diffusion components which are selected for immune cell differentiation through a gene-set enrichment analysis. The diffusion components are calculated from the diffusion map of the similarity matrix derived from the Gaussian kernel of the 10-nearest-neighbor Euclidean distance matrix from the 15-dimensional PCA projection of the normalized UMI gene counts [26].

**Fig 7:**
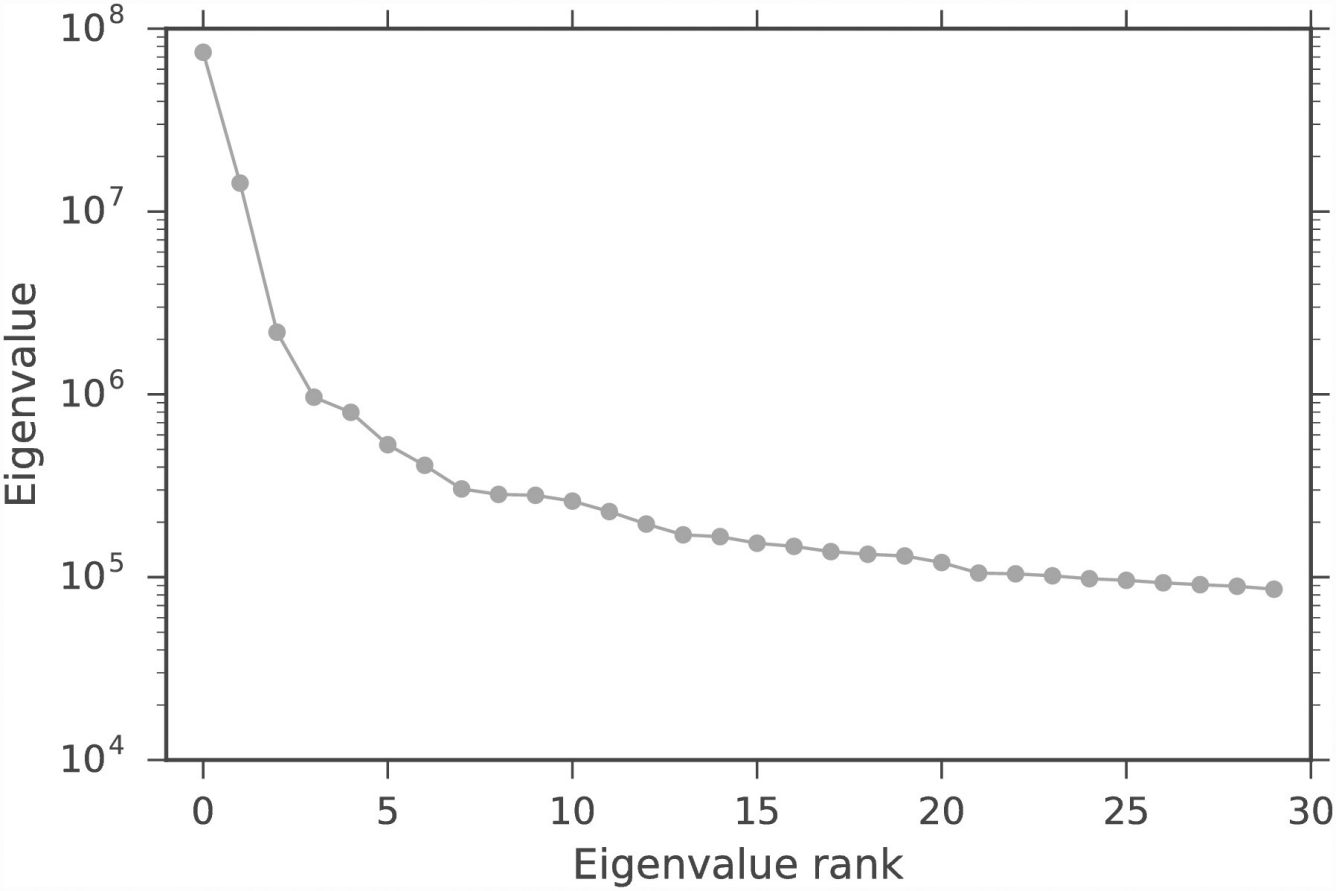
Eigenvalues for AA^*T*^, where A is the data matrix from the mouse bone marrow scRNA-Seq experiment [25] and the analysis workflow of [26]. “Elbows” are not as apparent as in Fig 1. The *wishbone* algorithm begins with a 15-dimensional PCA projection, so we choose the closest “elbow” at *k* = 14.

**Fig 8:**
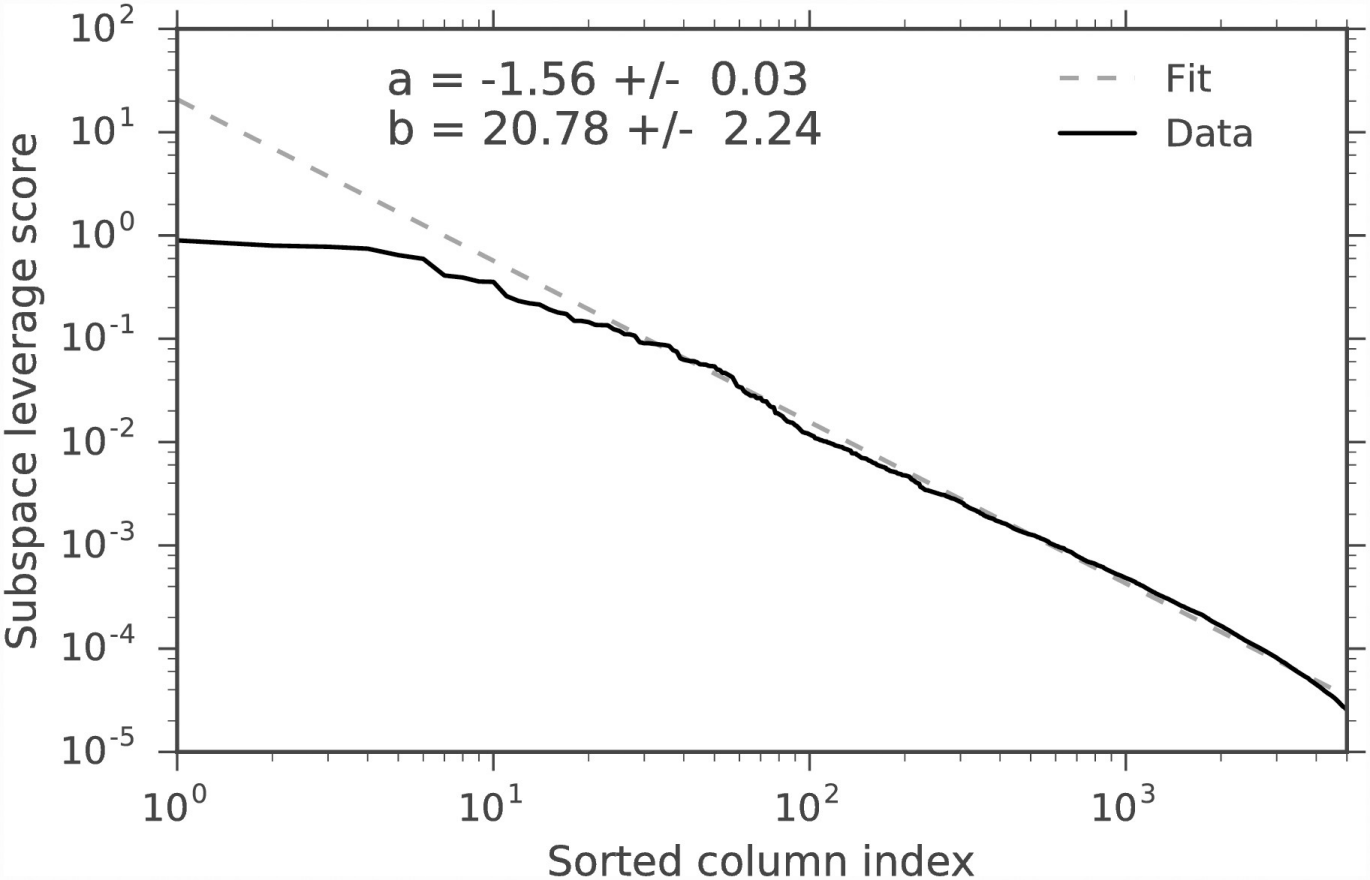
Power-law decay of *k* = 14 subspace leverage scores with sorted index for each column in A, where A is the data matrix from the mouse bone marrow scRNA-Seq experiment [25] and the analysis workflow of [26]. The fit is toScore = b × (Index)^*a*^. This shows that power-law decay is also a reasonable assumption for this dataset.

We choose *k* = 14 for the DCSS workflow by a combination of prior selection by the *wishbone* algorithm and the elbow method (Fig 7). The *wishbone* algorithm begins with a 15-dimensional PCA projection, and *k* = 14 is the closest (albeit slight) elbow to *k* = 15. We select an error tolerance of *ϵ* = 0.05. The DCSS algorithm ran in less than a minute. The rank-14 subspace leverage scores and the power-law fit for the top-scored 5, 000 genes are shown in Fig 8. The column submatrix **C** has 4, 693 genes, or approximately 31.4% of the total genes. These genes contain 90.4% of the UMI counts.

**Fig 9:**
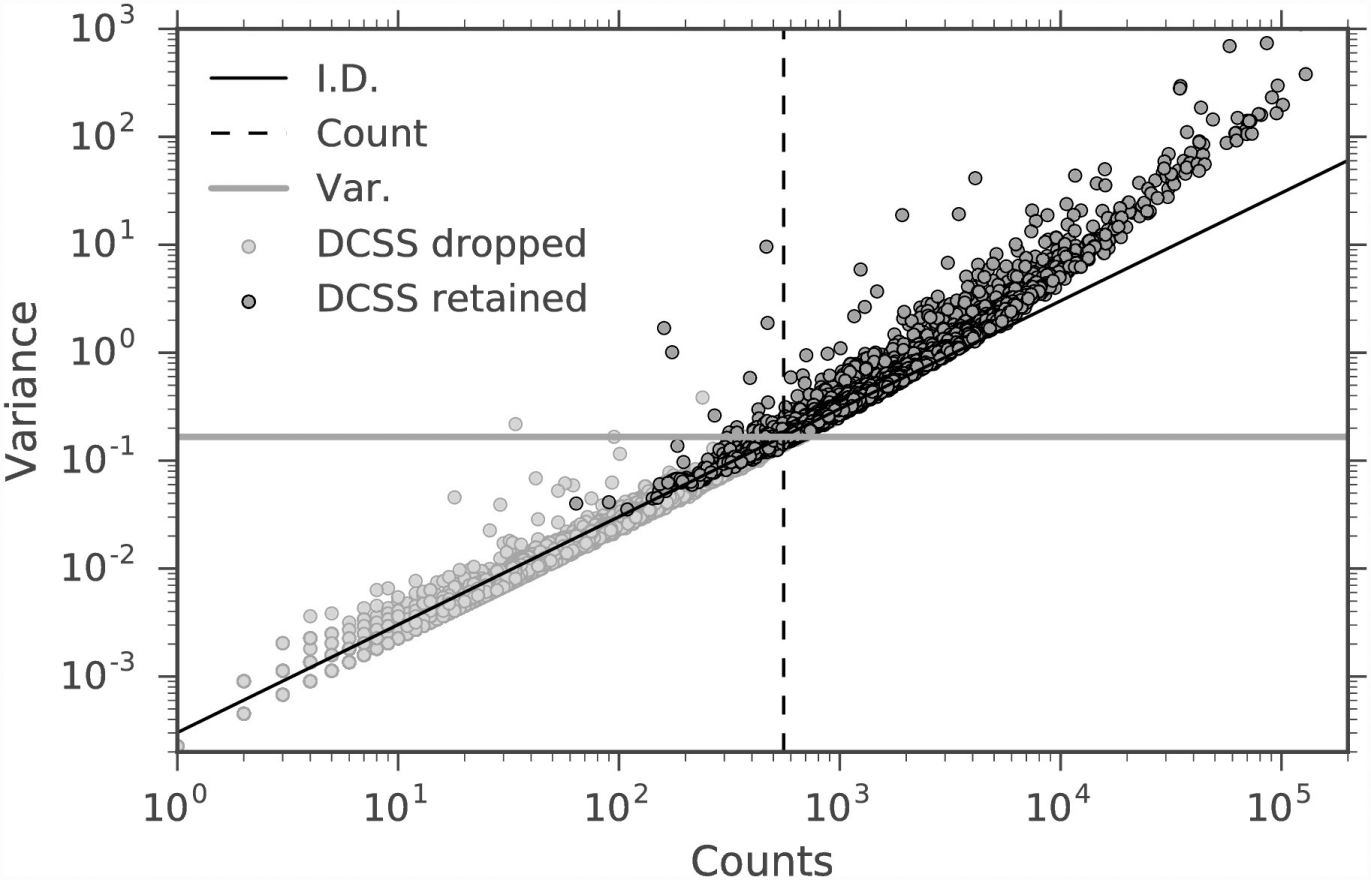
Count-variance plot for each column of A, where A is the data matrix from the mouse bone marrow scRNA-Seq experiment [25] and the analysis workflow of [26]. The color for each column represents whether the column is selected or not by *k* = 14, *ϵ* = 0.05 DCSS. The plot also shows the thresholds for count, variance, and index of dispersion with same number of selected columns as DCSS. The columns selected by DCSS are highly variable and have large counts, similar to Fig 3.

**Fig 10:**
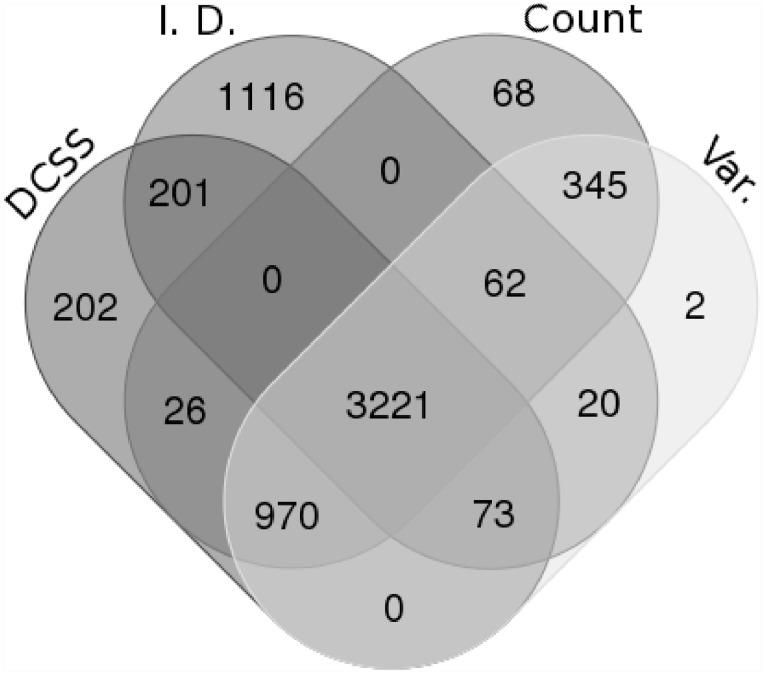
Venn diagram comparing the overlap between selected columns between *k* = 14, *ϵ* = 0.05 DCSS, count, variance, and index of dispersion thresholding on the data matrix from the mouse bone marrow scRNA-Seq experiment [25] and the analysis workflow of [26]. Figure tool credit: [33].

We compare DCSS thresholding with *k* = 14, *ϵ* = 0.05 to the three simple column thresholding methods with the same number of columns in Figs 9 and 10. The distribution of columns on the count-variance plots are qualitatively different between the mouse brain data (Fig 9) and the mouse bone marrow data (Fig 3). This difference is expected due to the differences between ECs and gene UMI counts. Although the index of dispersion method is more differentiated from the other methods on the mouse bone marrow dataset, the behavior of the DCSS method in relation to the simple column thresholding methods is similar between the datasets.

**Fig 11:**
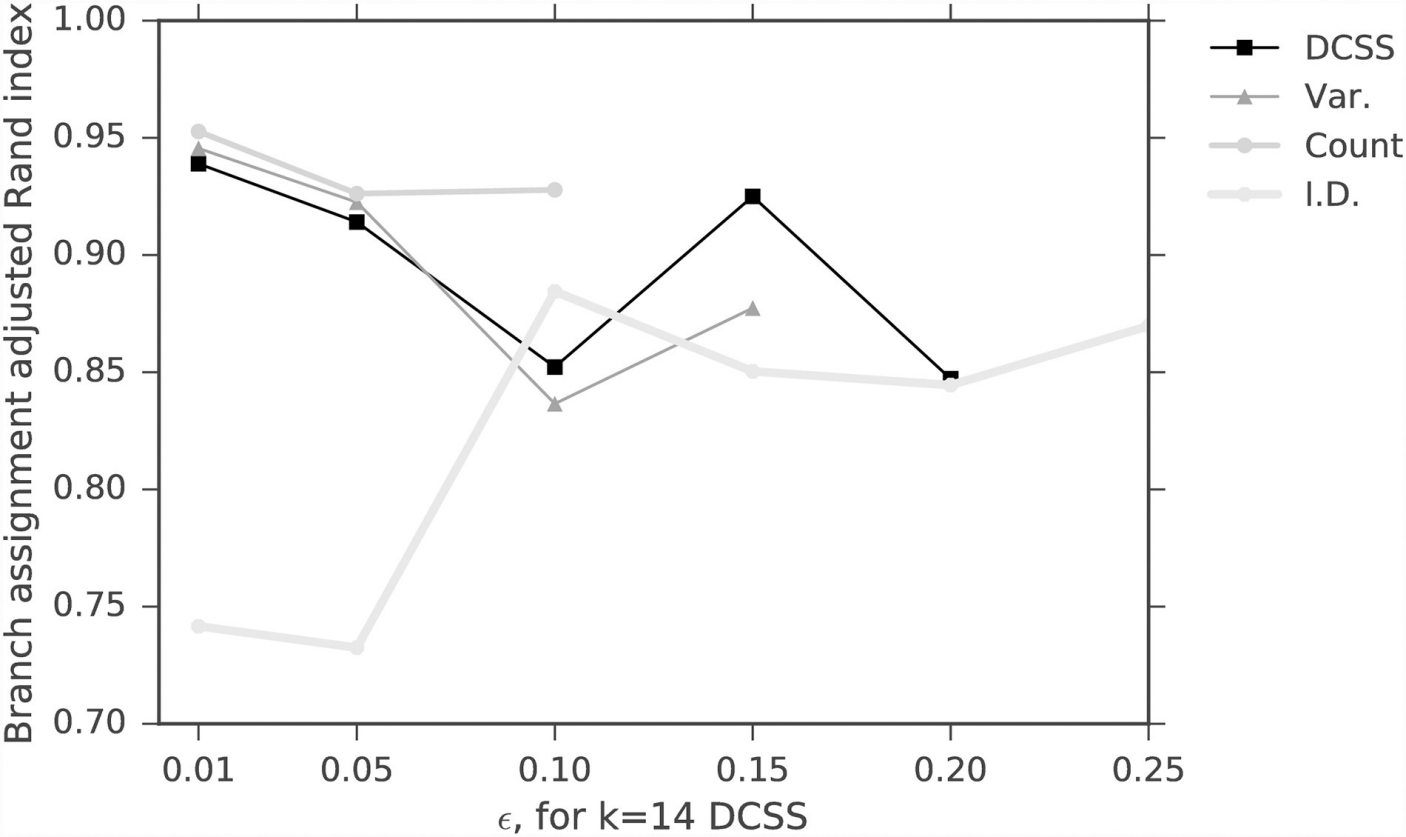
Average branch assignment ARI, varying the error tolerance *ϵ* with *k* = 14 for DCSS for the [25] and [26] dataset. We also threshold according to count, variance, and index of dispersion. The trend between error tolerance and average ARI is not as clear as in Fig 5.

**Fig 12:**
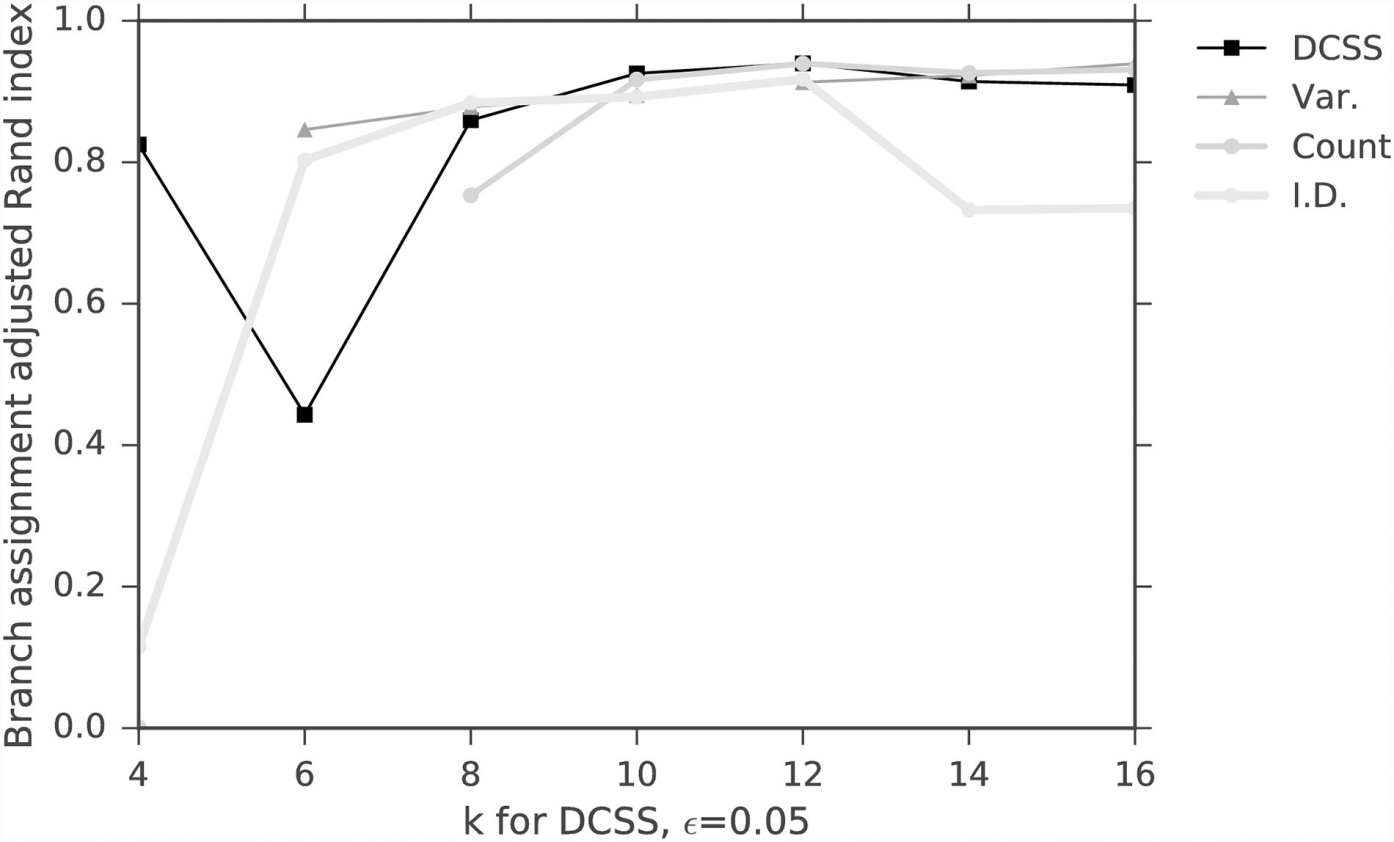
Average branch assignment ARI, varying the dimension *k* with fixed error tolerance *ϵ* = 0.05 for DCSS for the [25] and [26] dataset. We also threshold according to count, variance, and index of dispersion. The trend between dimension and average ARI is not as clear as in Fig 6.

We calculate the average *wishbone* classification ARI between the two workflows, again regarding the complete data workflow as the ground-truth. Since the *wishbone* algorithm utilizes random sampling, the average is over *T* = 10 *wishbone* branch assignments. The original *wishbone* analysis included only diffusion components 1 and 2. We additionally include diffusion component 4, since it is enriched for T cell differentiation according to the GSEA (see Table 2). For the mouse bone marrow dataset, *wishbone* assigns cells to three branches. [26] used the behavior of four markers (CD34, Gata1, Gata2, and Mpo) to verify that the three branches correspond to HSPCs, myeloid progenitors, and erythroid progenitors, and the behavior does not change with the inclusion of component 4. Figs 11 and 12 show the average branch assignment classification ARI for the workflow with the matrix **A** and the workflow with the column submatrix **C** for various *k*, *ϵ*, and also the three simple column thresholding methods with the same number of columns as the DCSS method for each *k*, *ϵ* point. Not all the thresholding methods successfully complete the *wishbone* workflow at large *ϵ*, due to the sensitivity of the diffusion component GSEA enrichment analysis, which we perform with keyword string matching. We find that for the *k* = 14, *ϵ* = 0.05 DCSS, 31.4% of the total genes give an average ARI of 0.91 for three branch assignments compared to the complete data; this supports our conclusion that only a small fraction of the gene expression profile from scRNA-Seq experiments may be necessary to obtain meaningful cell-type classifications.

**Table 2:** *wishbone* diffusion component 4, calculated from the complete data workflow, is enriched for T cell differentiation, according to the *wishbone* GSEA.

Gene ontology (GO) term	FDR q-value
Component 4, positive enrichment scores	
ribosome biogenesis (GO:0042254)	0.0
ribonucleoprotein complex biogenesis (GO:0022613)	0.0
rRNA processing (GO:0006364)	0.0
ncRNA metabolic process (GO:0034660)	0.0
rRNA metabolic process (GO:0016072)	0.0
Component 4, negative enrichment scores	
T cell differentiation (GO:0030217)	4.73E-03
second-messenger-mediated signaling (GO:0019932)	5.52E-03
regulation of actin cytoskeleton organization (GO:0032956)	5.76E-03
taxis (GO:0042330)	4.32E-03
regulation of actin filament-based process (GO:0032970)	5.04E-03

## Discussion

scRNA-Seq experiments allow researchers to probe the cell-specific heterogeneity in gene expression. Quality control and technical variability are significant challenges for scRNA-Seq experiments, and additionally the whole-genome expression profile is high-dimensional. In this note, we explore three simple column thresholding schemes– count, variance, and index of dispersion– and propose a novel application of a thresholding scheme—DCSS– to select informative features and control quality and technical variability. We prove a novel bound on the “closeness” of the DCSS data submatrix to the complete data matrix (Eq 9). This bound enlarges upon the existing set of error guarantees for DCSS [22] and provides a theoretical advantage over the three simple column thresholding schemes which have no similar guarantees. Other advantages of DCSS over simple column thresholding include sensitivity to collinearity of features and covariance of cells. Since scRNA-Seq experiments are frequently used to cluster and classify cells, we choose to evaluate these thresholding schemes for clustering and classification compared to the complete data and using the ARI.

We present two case studies, the first on mouse cortex and hippocampus scRNA-Seq [4, 24], and the second on mouse bone marrow scRNA-Seq [25, 26]. For the mouse brain, the data matrix is cells × ECs, and only an incredibly small fraction of the ECs are necessary to obtain neuron and non-neuron cell clusters. For the mouse bone marrow, the data matrix is cells × genes, and only a small fraction of the genes are necessary to obtain HSPC, myeloid progenitor, and erythroid progenitor branch assignments. For both case studies, DCSS performs similarly to the simple column thresholding schemes with the same number of columns, in that it reduces the low abundance genes, maintains the most variable and over-dispersed genes, and provides the additional benefit of theoretical guarantees. This supports our recommendation to use DCSS to control quality and technical variability. In both case studies, there is high similarity between the clustering computed with the complete expression profile and the reduced expression profile, suggesting that the clustering algorithms rely on a small subset of informative features.

## Supporting information

S1 File The supplementary material document contains supplementary figures, the mathematical definitions, a brief linear algebra review, and the proofs. (PDF)
