# Supplementary material for: Deterministic column subset selection for single-cell RNA-Seq
Source: PLoS One. 2019 Jan 25;14(1):e0210571. doi: 10.1371/journal.pone.0210571 (PMC6347249; doi:10.1371/journal.pone.0210571)
Supplement: S1 File — (PDF) [file pone.0210571.s001.pdf]

Deterministic column subset selection for single-cell RNA-Seq: Supplementary Material

Shannon R. McCurdy[1*], Vasilis Ntranos[2], Lior Pachter[3]

[1] California Institute for Quantitative Biosciences, University of California Berkeley, Berkeley, California, United States of America
[2] Department of Electrical Engineering and Computer Sciences, University of California Berkeley, Berkeley, California, United States of America
[3] Division of Biology and Biological Engineering, Department of Computing and Mathematical Sciences, California Institute of Technology, Pasadena, California, United States of America

* Corresponding author
E-mail: smccurdy@berkeley.edu (SRM)

A Additional figures

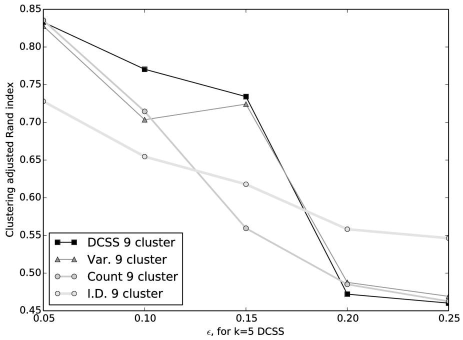

Figure A. Average spectral clustering ARI for nine clusters for DCSS, count, variance, and index of dispersion thresholding on the data matrix from the mouse cortex scRNA-Seq experiment [1] and the clustering workflow of [2]. We vary the error tolerance ϵ with $k = 5$ for DCSS. Increasing the error tolerance decreases the agreement between clusters.

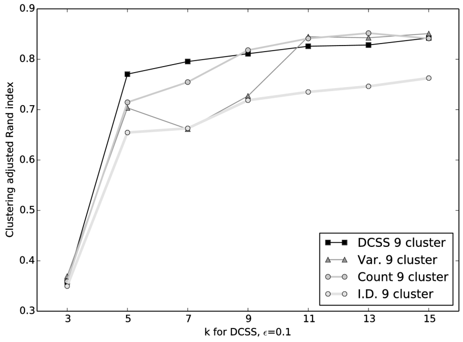

Figure B. Average spectral clustering ARI for nine clusters for DCSS, count, variance, and index of dispersion thresholding on the data matrix from the mouse cortex scRNA-Seq experiment [1] and the clustering workflow of [2]. We vary the dimension k with fixed error tolerance $\epsilon = 0.1$ for DCSS. Increasing the dimension increases the agreement between clusters.

B Brief linear algebra review [3]

The *singular value decomposition* (SVD) of any complex matrix $\mathbf{A}$ is $\mathbf{A} = \mathbf{U}\mathbf{\Sigma}\mathbf{V}^\dagger$, where $\mathbf{U}$ and $\mathbf{V}$ are square unitary matrices ($\mathbf{U}^\dagger\mathbf{U} = \mathbf{U}\mathbf{U}^\dagger = \mathbf{I}, \mathbf{V}^\dagger\mathbf{V} = \mathbf{V}\mathbf{V}^\dagger = \mathbf{I}$), $\mathbf{\Sigma}$ is a rectangular diagonal matrix with real non-negative non-increasingly ordered entries. $\mathbf{U}^\dagger$ is the complex conjugate and transpose of $\mathbf{U}$, and $\mathbf{I}$ is the identity matrix. The diagonal elements of $\mathbf{\Sigma}$ are called the *singular values*, and they are the positive square roots of the eigenvalues of both $\mathbf{A}\mathbf{A}^\dagger$ and $\mathbf{A}^\dagger\mathbf{A}$, which have eigenvectors $\mathbf{U}$ and $\mathbf{V}$, respectively. $\mathbf{U}$ and $\mathbf{V}$ are the *left* and *right singular vectors* of $\mathbf{A}$.

Defining $\mathbf{U}_k$ as the first k columns of $\mathbf{U}$ and analogously for $\mathbf{V}$, and $\mathbf{\Sigma}_k$ the square diagonal matrix with the first k entries of $\mathbf{\Sigma}$, then $\mathbf{A}_k = \mathbf{U}_k\mathbf{\Sigma}_k\mathbf{V}_k^\dagger$ is the rank-k SVD approximation to $\mathbf{A}$, and $\mathbf{T}_k = \mathbf{A}\mathbf{V}_k = \mathbf{U}_k\mathbf{\Sigma}_k$ is a rank-k SVD truncation of $\mathbf{A}$. Furthermore, we refer to matrix with only the last $n-k$ columns of $\mathbf{U}, \mathbf{V}$ and last $n-k$ entries in $\mathbf{\Sigma}$ as $\mathbf{U}_{\setminus k}, \mathbf{V}_{\setminus k}$, and $\mathbf{\Sigma}_{\setminus k}$.

The Moore-Penrose pseudo inverse of a rank r matrix $\mathbf{A}$ is given by $\mathbf{A}^+ = \mathbf{V}_r\mathbf{\Sigma}_r^{-1}\mathbf{U}_r^\dagger$.

The Frobenius norm $||\mathbf{A}||_F$ of a matrix $\mathbf{A}$ is given by $||\mathbf{A}||_F = \sqrt{\operatorname{tr}(\mathbf{A}\mathbf{A}^\dagger)}$. Recall that the trace has a cyclic property. The spectral norm $||\mathbf{A}||_2$ of a matrix $\mathbf{A}$ is given by the largest singular value of $\mathbf{A}$.

The Eckart-Young-Mirsky theorem [4] states that, for $\mathbf{A} = \mathbf{U}\mathbf{\Sigma}\mathbf{V}^\dagger$ the SVD of $\mathbf{A}$, and $\mathbf{B}$ any complex matrix with compatible dimension to $\mathbf{A}$ and rank $\leq k$,

$$\begin{aligned} \mathbf{A}_k &= \operatorname{argmin}_{\operatorname{rank}(\mathbf{B})\leq k}||\mathbf{A}-\mathbf{B}||_F \\ \min_{\operatorname{rank}(\mathbf{B})\leq k}||\mathbf{A}-\mathbf{B}||_F &= \sqrt{\operatorname{tr}(\mathbf{\Sigma}_{\setminus k}\mathbf{\Sigma}_{\setminus k}^T)}. \end{aligned} \tag{S1}$$

The minimizer $\mathbf{A}_k$ is unique if and only if $\sigma_{k+1} \neq \sigma_k$, where σ_i are the respective non-increasingly ordered singular values in $\mathbf{\Sigma}$.

A square complex matrix $\mathbf{F}$ is *Hermitian* if $\mathbf{F} = \mathbf{F}^\dagger$. Symmetric positive semi-definite (S.P.S.D) matrices are Hermitian matrices. The set of $n \times n$ Hermitian matrices is a real linear space. As such, it is possible to define a *partial ordering* (also called a Loewner partial ordering, denoted by $\preceq$) on the real linear space. One matrix is "greater" than another if their difference lies in the closed convex cone of S.P.S.D. matrices. Let $\mathbf{F}, \mathbf{G}$ be Hermitian and the same size, and $\mathbf{x}$ a complex vector of compatible dimension. Then,

$$\mathbf{F} \preceq \mathbf{G} \iff \mathbf{x}^\dagger\mathbf{F}\mathbf{x} \leq \mathbf{x}^\dagger\mathbf{G}\mathbf{x} \quad \forall\mathbf{x} \neq \mathbf{0}. \tag{S2}$$

A few simple consequences of the Loewner partial ordering are as follows. If $\mathbf{F}$ is Hermitian and S.P.S.D., then $\mathbb{0} \preceq \mathbf{F}$, where $\mathbb{0}$ is the zero matrix.

If $\mathbf{F}$ is Hermitian with smallest and largest eigenvalues $\lambda_{\min}(\mathbf{F}), \lambda_{\max}(\mathbf{F})$, respectively, then,

$$\lambda_{\min}(\mathbf{F})\mathbf{I} \preceq \mathbf{F} \preceq \lambda_{\max}(\mathbf{F})\mathbf{I}. \tag{S3}$$

Let $\mathbf{F}, \mathbf{G}$ be Hermitian and the same size, and let $\mathbf{H}$ be any complex rectangular matrix of compatible dimension. The *conjugation rule* is,

$$\text{If } \mathbf{F} \preceq \mathbf{G}\text{, then } \mathbf{H}\mathbf{F}\mathbf{H}^\dagger \preceq \mathbf{H}\mathbf{G}\mathbf{H}^\dagger. \tag{S4}$$

In addition, let $\lambda_i(\mathbf{F})$ and $\lambda_i(\mathbf{G})$ be the non-decreasingly ordered eigenvalues of $\mathbf{F}, \mathbf{G}$. Then,

$$\text{If } \mathbf{F} \preceq \mathbf{G}\text{, then } \forall i,\ \lambda_i(\mathbf{F}) \leq \lambda_i(\mathbf{G}). \tag{S5}$$

Since the trace of a matrix $\mathbf{F}$ is the sum of its eigenvalues, $\operatorname{tr}\mathbf{F} = \sum_i \lambda_i(\mathbf{F})$, and the Loewner ordering implies the ordering of eigenvalues (Eq S5), the Loewner ordering also implies the ordering of their sum,

$$\text{If } \mathbf{F} \preceq \mathbf{G}, \text{ then } \operatorname{tr}\mathbf{F} \leq \operatorname{tr}\mathbf{G}. \tag{S6}$$

Let $\mathbf{F}_1, \mathbf{G}_1, \mathbf{F}_2, \mathbf{G}_2$ be Hermitian and the same size. Then if $\mathbf{F}_1 \preceq \mathbf{G}_1$ and $\mathbf{F}_2 \preceq \mathbf{G}_2$, then

$$\mathbf{F}_1 + \mathbf{F}_2 \preceq \mathbf{G}_1 + \mathbf{G}_2. \tag{S7}$$

As a simple consequence of Eq S2, consider the real matrices $\mathbf{F}\mathbf{F}^T$ and $\mathbf{G}\mathbf{G}^T$, and the vector $\mathbf{x}$ which has a one in row i and a minus one in row j, and zeros elsewhere. The Euclidean distance between rows i, j with respect to $\mathbf{G}$ is $d_{i,j}(\mathbf{G})$:

$$d_{i,j}(\mathbf{G}) = \mathbf{x}^T\mathbf{G}\mathbf{G}^T\mathbf{x}. \tag{S8}$$

Thus, if $\mathbf{F}\mathbf{F}^T \preceq \mathbf{G}\mathbf{G}^T$, by Eq S2 with appropriate vectors, $d_{i,j}(\mathbf{F}) \leq d_{i,j}(\mathbf{G}) \forall i, j$.

Furthermore, let $\mathbf{F}$ be Hermitian and dimension n, $\mathbf{U}_k$ be a semi-orthogonal rectangular matrix ($\mathbf{U}_k^\dagger \mathbf{U}_k = \mathbf{I}$) of compatible dimension $n \times k$, $1 \leq k \leq n$, and $\lambda_i(\mathbf{M})$ refer to the non-decreasingly ordered eigenvalues of a matrix $\mathbf{M}$. Then the upper bound of the *Poincaré separation theorem* states,

$$\lambda_i(\mathbf{U}_k^\dagger \mathbf{F} \mathbf{U}_k) \preceq \lambda_{n-k+i}(\mathbf{F}) \quad i = 1, \ldots, k. \tag{S9}$$

We will also use the von Neumann trace inequality. Let $\mathbf{F}, \mathbf{G}$ be complex matrices of compatible dimension and minimum dimension n. Let $\sigma_i(\mathbf{F}), \sigma_i(\mathbf{G})$ be the respective non-increasingly ordered singular values. Then

$$Re(\operatorname{tr}\mathbf{F}\mathbf{G}^\dagger) \leq \sum_{i=1}^{n} \sigma_i(\mathbf{F})\sigma_i(\mathbf{G}). \tag{S10}$$

C Proof of Eq 2

Eq 2 is a generalization of Lemma 2 in [5]. The proof is as follows. The minimum norm solution to the least-squares minimization problem $\min_{\mathbf{x}} ||\mathbf{A}_k\mathbf{x} - \mathbf{a}_i||_2^2$ is,

$$\hat{\mathbf{x}} = \mathbf{A}_k^+ \mathbf{a}_i = \mathbf{V}_k \mathbf{\Sigma}_k^{-1} \mathbf{U}_k^\dagger \mathbf{a}_i. \tag{S11}$$

And, by definition,

$$||\hat{\mathbf{x}}||_2^2 = \mathbf{a}_i^T \mathbf{U}_k \mathbf{\Sigma}_k^{-1} \mathbf{V}_k^\dagger \mathbf{V}_k \mathbf{\Sigma}_k^{-1} \mathbf{U}_k^\dagger \mathbf{a}_i = \mathbf{a}_i^T \mathbf{U}_k \mathbf{\Sigma}_k^{-2} \mathbf{U}_k^\dagger \mathbf{a}_i = \tau_i(\mathbf{A}_k). \tag{S12}$$

D Proof of Eq 9

The upper bound (Eq 9) in Theorem 1 follows from the fact that $\mathbb{0} \preceq \mathbf{I} - \mathbf{S}\mathbf{S}^T$ and the conjugation rule (Eq S4),

$$\mathbb{0} \quad \preceq \quad \mathbf{A}(\mathbf{I} - \mathbf{S}\mathbf{S}^T)\mathbf{A}^T = \mathbf{A}\mathbf{A}^T - \mathbf{C}\mathbf{C}^T. \tag{S13}$$

This upper bound is true for any column selection of $\mathbf{A}$. A second application of the conjugation rule gives the upper bound in Eq 9.

For the lower bound (Eq 9), consider the quantity $\mathbf{Y} = \mathbf{\Sigma}_k^{-1}\mathbf{U}_k^T\mathbf{A}(\mathbf{I} - \mathbf{S}\mathbf{S}^T)\mathbf{A}^T\mathbf{U}_k\mathbf{\Sigma}_k^{-1} = \mathbf{V_k}^T(\mathbf{I} - \mathbf{S}\mathbf{S}^T)\mathbf{V_k}$. By the conjugation rule (Eq

S4) on Eq S13, $\mathbb{0} \preceq \mathbf{Y}$, so $\mathbf{Y}$ is S.P.S.D. By the construction of DCSS (Eq 3) $\operatorname{tr}\mathbf{Y} = \sum_{i \notin \Theta}\sum_{l=1}^{k} V_{il}^2 = \tilde{\epsilon} < \epsilon$, and because $\mathbf{Y}$ is S.P.S.D., $\lambda_{\max}(\mathbf{Y}) \leq \operatorname{tr}\mathbf{Y}$. By Eq S3 and the previous facts, $\mathbf{Y} \preceq \lambda_{\max}(\mathbf{Y})\mathbf{I} \preceq \epsilon\mathbf{I}$. As a result of the conjugation rule applied to this upper bound,

$$\begin{aligned} \mathbf{U}_k\mathbf{\Sigma}_k\mathbf{Y}\mathbf{\Sigma}_k\mathbf{U}_k^T = \mathbf{A}_k\mathbf{A}_k^T - \mathbf{U}_k\mathbf{U}_k^T\mathbf{C}\mathbf{C}^T\mathbf{U}_k\mathbf{U}_k^T &\preceq \epsilon\mathbf{A}_k\mathbf{A}_k^T \\ (1-\epsilon)\mathbf{A}_k\mathbf{A}_k^T &\preceq \mathbf{U}_k\mathbf{U}_k^T\mathbf{C}\mathbf{C}^T\mathbf{U}_k\mathbf{U}_k^T, \end{aligned} \tag{S14}$$

providing the lower bound of Eq 9.

For Eq 10, the lower bound of Eq 9 implies,

$$(1-\epsilon)\operatorname{tr}\mathbf{A}_k\mathbf{A}_k^T \quad \leq \quad \operatorname{tr}\mathbf{U}_k^T\mathbf{C}\mathbf{C}^T\mathbf{U}_k, \tag{S15}$$

by Eq S6 and the cyclic property of the trace. Similarly, Eq S13 implies $\operatorname{tr}\mathbf{C}\mathbf{C}^T \leq \operatorname{tr}\mathbf{A}\mathbf{A}^T$. Since $\mathbf{U}_k$ is semi-orthogonal ($\mathbf{U}_k^T\mathbf{U}_k = \mathbf{I}$), by Eq S9, every ordered eigenvalue of $\mathbf{U}_k^T\mathbf{C}\mathbf{C}^T\mathbf{U}_k$ is smaller than its counterpart ordered eigenvalue of $\mathbf{C}\mathbf{C}^T$. Since the trace is the sum of eigenvalues, this implies Eq 10,

$$(1-\epsilon)\operatorname{tr}\mathbf{A}_k\mathbf{A}_k^T \leq \operatorname{tr}\mathbf{U}_k^T\mathbf{C}\mathbf{C}^T\mathbf{U}_k \leq \operatorname{tr}\mathbf{C}\mathbf{C}^T \leq \operatorname{tr}\mathbf{A}\mathbf{A}^T. \tag{S16}$$

Note that if $\mathbf{A}$ is full rank and $k = rank(\mathbf{A}) = n$, then Eq 9 becomes,

$$(1-\epsilon)\mathbf{A}\mathbf{A}^T \quad \preceq \quad \mathbf{C}\mathbf{C}^T \preceq \mathbf{A}\mathbf{A}^T. \tag{S17}$$

E Proof of Eq 11 for random sampling

The following theorem pertains to a new spectral bound for the square $\mathbf{C}$ selected by rank-k subspace leverage scores and the random sampling procedure from [6]).

Theorem 1. *Let $\mathbf{A} \in \mathbb{R}^{n \times d}$ be a matrix of at least rank k and $\tau_i(\mathbf{A}_k)$ be defined as in Eq 1. Construct $\mathbf{C}$ by sampling t columns of $\mathbf{A}$, reweighted to $\frac{1}{\sqrt{tp_i}}\mathbf{a}_i$, with probability $p_i = (\tau_i(\mathbf{A}_k) + \gamma\mathbb{1}(\tau_i(\mathbf{A}_k) = 0))/(\sum_{i=1}^{d} p_i)$, where $\mathbb{1}()$ is the indicator function and γ is a small, positive, non-zero number $\gamma = \min_{\tau_i(\mathbf{A}_k)>0}(\tau_i(\mathbf{A}_k))$. Let $m = \sum_{i=1}^{d}\mathbb{1}(\tau_i(\mathbf{A}_k) = 0)$, $\sum_{i=1}^{d} p_i = k + m\gamma$. If the number of selected columns $t \geq \frac{2}{\epsilon^2}(k + m\gamma)\left(1 + \frac{1}{3}\epsilon\right)\ln\left(\frac{16k}{\delta}\right)$, then with probability $1-\delta$, the matrix $\mathbf{C}$ satisfies:*

$$(1-\epsilon)\mathbf{A}_k\mathbf{A}_k^T \preceq \mathbf{U}_k\mathbf{U}_k^T\mathbf{C}\mathbf{C}^T\mathbf{U}_k\mathbf{U}_k^T \preceq (1+\epsilon)\mathbf{A}_k\mathbf{A}_k^T. \tag{S18}$$

If $\mathbf{A}$ is full rank and $k = rank(\mathbf{A}) = n$, then Eq S18 becomes,

$$(1-\epsilon)\mathbf{A}\mathbf{A}^T \preceq \mathbf{C}\mathbf{C}^T \preceq (1+\epsilon)\mathbf{A}\mathbf{A}^T. \tag{S19}$$

The proof of Theorem 1 is similar in structure to Theorem 3 in [7]. Theorem 3 in [7] pertains to a different type of leverage score.

Consider the quantity $\mathbf{Y} = \mathbf{\Sigma}_k^{-1}\mathbf{U}_k^T(\mathbf{C}\mathbf{C}^T - \mathbf{A}\mathbf{A}^T)\mathbf{U}_k\mathbf{\Sigma}_k^{-1}$. Note the sign change from Section Proof of Eq 9. This can be rewritten as,

$$\begin{aligned} \mathbf{Y} &= \sum_{j=1}^{t}\mathbf{\Sigma}_k^{-1}\mathbf{U}_k^T(\mathbf{c}_j\mathbf{c}_j^T - \tfrac{1}{t}\mathbf{A}\mathbf{A}^T)\mathbf{U}_k\mathbf{\Sigma}_k^{-1} \\ \mathbf{Y} &= \sum_{j=1}^{t}\mathbf{X}_j, \\ \forall j, (\mathbf{X}_j)_i &= \tfrac{1}{t}\mathbf{\Sigma}_k^{-1}\mathbf{U}_k^T(\tfrac{1}{p_i}\mathbf{a}_i\mathbf{a}_i^T - \mathbf{A}\mathbf{A}^T)\mathbf{U}_k\mathbf{\Sigma}_k^{-1} \quad \text{with categorical probability } p_i. \end{aligned} \tag{S20}$$

If $||\mathbf{Y}||_2 \leq \epsilon$, then $-\epsilon\mathbf{I} \preceq \mathbf{Y} \preceq \epsilon\mathbf{I}$, and Eq S18 follows from this and the definition of $\mathbf{Y}$. Thus, the proof of Eq S18 relies on showing that $||\mathbf{Y}||_2 \leq \epsilon$. We use an intrinsic dimension matrix Bernstein inequality ([8] , Theorem 7.3.1), specialized to Hermitian matrices, to show that $||\mathbf{Y}||_2$ is small with high probability. The Bernstein inequality requires that, for a finite sequence $\mathbf{Y} = \sum_{j=1}^{t} \mathbf{X}_j$ of random Hermitian matrices $\mathbf{X}_j$ of the same size,

1. $\forall j, \mathbb{E}(\mathbf{X}_j) = 0$,
2. $\forall j, ||\mathbf{X}_j||_2 \leq L$,
3. and that $\sum_j \mathbb{E}(\mathbf{X}_j\mathbf{X}_j^T) \preceq \mathbf{V}$.

Then, for $\epsilon \geq \sqrt{||\mathbf{V}||_2} + L/3$,

$$\mathbf{P}(||\mathbf{Y}||_2 \geq \epsilon) \leq 8\frac{\operatorname{tr}\mathbf{V}}{||\mathbf{V}||_2}\exp\left(-\frac{\epsilon^2/2}{\epsilon L/3 + ||\mathbf{V}||_2}\right). \tag{S21}$$

Requirement 1 is satisfied because,

$$\mathbb{E}(\mathbf{X}_j) = \sum_{i=1}^{d} p_i(\mathbf{X}_j)_i = \tfrac{1}{t}\mathbf{\Sigma}_k^{-1}\mathbf{U}_k^T(\sum_{j=1}^{d}\mathbf{a}_i\mathbf{a}_i^T - \mathbf{A}\mathbf{A}^T)\mathbf{U}_k\mathbf{\Sigma}_k^{-1} = 0. \tag{S22}$$

To show that requirement 2 is satisfied, we need the following fact:

$$\mathbf{\Sigma}_k^{-1}\mathbf{U}_k^T\mathbf{a}_i\mathbf{a}_i^T\mathbf{U}_k\mathbf{\Sigma}_k^{-1} \preceq \tau_i(\mathbf{A}_k)\mathbf{I}. \tag{S23}$$

Eq S23 follows from the fact that for all $\mathbf{y} \in \mathbb{R}^k$,

$$\mathbf{y}^T\mathbf{U}_k\mathbf{\Sigma}_k^{-1}\mathbf{U}_k^T\mathbf{a}_i\mathbf{a}_i^T\mathbf{U}_k\mathbf{\Sigma}_k^{-1}\mathbf{U}_k^T\mathbf{y} = \operatorname{tr}\left(\left(\mathbf{y}\mathbf{y}^T\right)\left(\mathbf{U}_k\mathbf{\Sigma}_k^{-1}\mathbf{U}_k^T\mathbf{a}_i\mathbf{a}_i^T\mathbf{U}_k\mathbf{\Sigma}_k^{-1}\mathbf{U}_k^T\right)\right) \leq \tau_i(\mathbf{A}_k)\mathbf{y}^T\mathbf{y}.$$

where the inequality comes from the Von Neumann trace inequality (Eq S10) applied to the product of two rank 1 matrices. Using Eq S23 in the definition of $\mathbf{X}_i$ gives,

$$\begin{aligned}
\mathbf{X}_j = \tfrac{1}{tp_i}\mathbf{\Sigma}_k^{-1}\mathbf{U}_k^T\mathbf{a}_i\mathbf{a}_i^T\mathbf{U}_k\mathbf{\Sigma}_k^{-1} - \tfrac{1}{t}\mathbf{I} &\preceq \tfrac{1}{tp_i}\tau_i(\mathbf{A}_k)\mathbf{I} - \tfrac{1}{t}\mathbf{I} \\
&= \tfrac{(k+m\gamma)\tau_i(\mathbf{A}_k)}{t(\tau_i(\mathbf{A}_k)+\gamma\mathbb{1}(\tau_i(\mathbf{A}_k)=0))}\mathbf{I} - \tfrac{1}{t}\mathbf{I} \\
&\preceq \tfrac{k+m\gamma}{t}\mathbf{I},
\end{aligned} \tag{S24}$$

and $||\mathbf{X}_j||_2 \leq L = \frac{k+m\gamma}{t}$ follows immediately.

To show that requirement 3 is satisfied, we compute directly,

$$\begin{aligned}
\mathbb{E}(\mathbf{Y}^2) &= t\mathbb{E}(\mathbf{X}_j\mathbf{X}_j^T) \\
&= t\sum_{i=1}^{d} p_i\Big(\left(\tfrac{1}{t}\mathbf{\Sigma}_k^{-1}\mathbf{U}_k^T(\tfrac{1}{p_i}\mathbf{a}_i\mathbf{a}_i^T - \mathbf{A}\mathbf{A}^T)\mathbf{U}_k\mathbf{\Sigma}_k^{-1}\right) \\
&\quad \cdot \left(\tfrac{1}{t}\mathbf{\Sigma}_k^{-1}\mathbf{U}_k^T(\tfrac{1}{p_i}\mathbf{a}_i\mathbf{a}_i^T - \mathbf{A}\mathbf{A}^T)\mathbf{U}_k\mathbf{\Sigma}_k^{-1}\right)\Big) \\
&= t\sum_{i=1}^{d} p_i\left(\left(\tfrac{1}{t}\mathbf{\Sigma}_k^{-1}\mathbf{U}_k^T(\tfrac{1}{p_i}\mathbf{a}_i\mathbf{a}_i^T - \mathbf{A}\mathbf{A}^T)\mathbf{U}_k\mathbf{\Sigma}_k^{-1}\right)\left(\tfrac{1}{tp_i}\mathbf{\Sigma}_k^{-1}\mathbf{U}_k^T\mathbf{a}_i\mathbf{a}_i^T\mathbf{U}_k\mathbf{\Sigma}_k^{-1}\right)\right) \\
&= t\sum_{i=1}^{d} p_i\left(\tfrac{1}{t^2p_i^2}\mathbf{\Sigma}_k^{-1}\mathbf{U}_k^T\mathbf{a}_i\mathbf{a}_i^T\mathbf{U}_k\mathbf{\Sigma}_k^{-2}\mathbf{U}_k^T\mathbf{a}_i\mathbf{a}_i^T\mathbf{U}_k\mathbf{\Sigma}_k^{-1}\right) - \tfrac{1}{t}\mathbf{I} \\
&\preceq \sum_{i=1}^{d}\left(\tfrac{1}{tp_i}\mathbf{\Sigma}_k^{-1}\mathbf{U}_k^T\mathbf{a}_i\mathbf{a}_i^T\mathbf{U}_k\mathbf{\Sigma}_k^{-1}\tau_i(\mathbf{A}_k)\mathbf{I}\right) - \tfrac{1}{t}\mathbf{I}
\end{aligned}$$

$$\preceq \quad \tfrac{k+m\gamma}{t} \sum_{i=1}^{d} \left(\boldsymbol{\Sigma}_k^{-1} \mathbf{U}_k^T \mathbf{a}_i \mathbf{a}_i^T \mathbf{U}_k \boldsymbol{\Sigma}_k^{-1} \right) = \tfrac{k+m\gamma}{t} \mathbf{I} = \mathbf{V}. \tag{S25}$$

It follows immediately that $||\mathbf{V}||_2 = \frac{k+m\gamma}{t}$ and $\operatorname{tr} \mathbf{V} = \frac{k(k+m\gamma)}{t}$.

Then, for $\epsilon \geq \sqrt{\frac{k+m\gamma}{t}} + \frac{k+m\gamma}{3t}$,

$$\mathbf{P}(||\mathbf{Y}||_2 \geq \epsilon) \leq 8k \exp\left(-\tfrac{t\epsilon^2/2}{(k+m\gamma)(\epsilon/3+1)} \right) \leq \tfrac{1}{2}\delta. \tag{S26}$$

Solving for t as a function of ϵ, δ, and γ gives,

$$t \geq \tfrac{2}{\epsilon^2}(k+m\gamma)\left(1+\tfrac{1}{3}\epsilon\right)\ln\left(\tfrac{16k}{\delta}\right). \tag{S27}$$

Eq S18 also holds for $\mathbf{C}$ selected by the DCSS algorithm, as a consequence of Eq 9. Thus DCSS selects fewer columns with the same accuracy for power-law decay for Eq S18 when $|\Theta| < t$.

References

1. Zeisel A, Muñoz-Manchado AB, Codeluppi S, Lönnerberg P, Manno GL, Juréus A, et al. Cell types in the mouse cortex and hippocampus revealed by single-cell RNA-seq. Science. 2015;347(6226):1138–1142. doi:10.1126/science.aaa1934.

2. Ntranos V, Kamath GM, Zhang JM, Pachter L, Tse DN. Fast and accurate single-cell RNA-seq analysis by clustering of transcript-compatibility counts. Genome Biology. 2016;17(1):1–14. doi:10.1186/s13059-016-0970-8.

3. Horn RA, Johnson CR. Matrix analysis. 2nd ed. New York: Cambridge University Press; 2013.

4. Eckart C, Young G. The approximation of one matrix by another of lower rank. Psychometrika. 1936;1:211–218. doi:10.1007/BF02288367.

5. Cohen MB, Lee YT, Musco C, Musco C, Peng R, Sidford A. Uniform Sampling for Matrix Approximation. In: Proceedings of the 2015 Conference on Innovations in Theoretical Computer Science. ITCS '15. New York, NY, USA: ACM; 2015. p. 181–190. Available from: `http://doi.acm.org/10.1145/2688073.2688113`.

6. Drineas P, Mahoney MW, Muthukrishnan S. Relative-Error CUR Matrix Decompositions. SIAM J Matrix Anal Appl. 2008;30(2):844–881. doi:10.1137/07070471X.

7. Cohen MB, Musco C, Musco C. Input Sparsity Time Low-rank Approximation via Ridge Leverage Score Sampling. In: Proceedings of the Twenty-Eighth Annual ACM-SIAM Symposium on Discrete Algorithms. SODA '17. Philadelphia, PA, USA: Society for Industrial and Applied Mathematics; 2017. p. 1758–1777. Available from: `http://dl.acm.org/citation.cfm?id=3039686.3039801`.

8. Tropp JA. An Introduction to Matrix Concentration Inequalities. Found Trends Mach Learn. 2015;8(1-2):1–230. doi:10.1561/2200000048.
